# Bacterial chemolithoautotrophy in ultramafic plumes along the Mid-Atlantic Ridge

**DOI:** 10.1093/ismejo/wrae165

**Published:** 2024-08-20

**Authors:** Bledina Dede, Eoghan P Reeves, Maren Walter, Wolfgang Bach, Rudolf Amann, Anke Meyerdierks

**Affiliations:** Max Planck Institute for Marine Microbiology, Bremen, Germany; Ecologie Systématique Evolution, CNRS, Université Paris-Saclay, AgroParisTech, Gif-sur-Yvette, France; Department of Earth Science and Centre for Deep Sea Research, University of Bergen, Bergen, Norway; Institute of Environmental Physics, University of Bremen, Bremen, Germany; MARUM, Center for Marine Environmental Sciences, University of Bremen, Bremen, Germany; MARUM, Center for Marine Environmental Sciences, University of Bremen, Bremen, Germany; Geoscience Department, University of Bremen, Bremen, Germany; Max Planck Institute for Marine Microbiology, Bremen, Germany; Max Planck Institute for Marine Microbiology, Bremen, Germany

**Keywords:** carbon monoxide oxidation, hydrogen oxidation, microbial ecology, metagenome analysis, hydrothermal vent, Sulfurimonas, SAR202

## Abstract

Hydrothermal vent systems release reduced chemical compounds that act as an important energy source in the deep sea. Chemolithoautotrophic microbes inhabiting hydrothermal plumes oxidize these compounds, in particular, hydrogen and reduced sulfur, to obtain the energy required for CO_2_ fixation. Here, we analysed the planktonic communities of four hydrothermal systems located along the Mid-Atlantic Ridge: Irinovskoe, Semenov-2, Logatchev-1, and Ashadze-2, by combining long-read 16S rRNA gene analysis, fluorescence in situ hybridization, meta-omics, and thermodynamic calculations. *Sulfurimonas* and SUP05 dominated the microbial communities in these hydrothermal plumes. Investigation of *Sulfurimonas* and SUP05 MAGs, and their gene transcription in plumes indicated a niche partitioning driven by hydrogen and sulfur. In addition to sulfur and hydrogen oxidation, a novel SAR202 clade inhabiting the plume, here referred to as genus *Carboxydicoccus,* harbours the capability for CO oxidation and CO_2_ fixation via reverse TCA cycle. Both pathways were also highly transcribed in other hydrogen-rich plumes, including the Von Damm vent field. *Carboxydicoccus profundi* reached up to 4% relative abundance (1.0 x 10^3^ cell ml^- 1^) in Irinovskoe non-buoyant plume and was also abundant in non-hydrothermally influenced deep-sea metagenomes (up to 5 RPKM). Therefore, CO, which is probably not sourced from the hydrothermal fluids (1.9–5.8 μM), but rather from biological activities within the rising fluid, may serve as a significant energy source in hydrothermal plumes. Taken together, this study sheds light on the chemolithoautotrophic potential of the bacterial community in Mid-Atlantic Ridge plumes.

## Introduction

Hydrothermal vent fields release chemically diverse fluids into the open ocean [1]. These hot fluids mix with the surrounding cold oxygenated seawater and rise until a point of neutral buoyancy, whereat laterally spreading hydrothermal plumes are formed. Such plumes are rich in reduced chemical compounds, which can provide high-energy yields [2–4] and support microbial carbon fixation [5, 6]. In ultramafic-hosted and basalt-hosted hydrothermal plumes, microbial communities are dominated by *Sulfurimonas* [7, 8] and SUP05 [2, 9–12] that oxidize reduced sulfur compounds and H_2_. However, other reduced molecules available in these systems may also be playing a role in shaping microbial communities, such as CH_4*,*_ whose influence has been demonstrated in different ocean basins [13], and Fe^2+^, which has been suggested by modelling and statistical analysis [9, 14] to yield energy through oxidation. A less studied reduced molecule, which may also fuel growth in hydrothermal plumes, is carbon monoxide (CO) – known to be present in low amounts in H_2_-rich hydrothermal fluids [15, 16].

Dissolved CO has been shown to serve as an energy and carbon source for microbes in the surface ocean [17, 18]. Diverse aerobic CO-oxidizing microbial taxa inhabit the oceans and have been categorized into two groups, the carboxydotrophs and the carboxydovores [19]. Carboxydotrophs utilize CO as a sole energy and carbon source [19–23]. In such organisms, CO oxidation is catalyzed by a low affinity molybdenum-copper CO-dehydrogenase (CODH), classified as form I [24, 25]. The energy produced from CO oxidation supports ATP synthesis and carbon fixation via the Calvin–Benson–Bassham (CBB) cycle [19]. In contrast, carboxydovores use CO only for energy gain and require organic carbon for growth as they lack a complete CBB cycle [19, 26]. Carboxydovores have been reported to harbour, in addition to form I, a related form II CODH [19, 26]. Many theories regarding this metabolism have been raised [26, 27]. Recently, cultivation experiments were used to show that CO oxidation enables carboxydovores to survive in substrate-limited environments [28].

CO may also support chemolithoautotrophic life in the deep sea. Despite reduced CO concentrations below 10 m depth (<0.5 nM) [29], the number of CODH encoding genes increases with depth [30]. In almost all known hydrothermal systems, endmember CO concentrations are much higher, with values <0.05 μM reported in very H_2_-depleted fluids from the Manus Basin [31], 0.22–7.4 μM in H_2_-rich, bare-rock hosted systems [16], to up to 225 μM recently reported in the organic- and H_2_-rich fluids of the Guaymas Basin [32]. High concentrations of CO could allow for the growth of both carboxydotrophic and carboxydovoric bacteria in the near vent environment. A potential candidate for this metabolism is the deep-sea cosmopolitan SAR202 clade, because CODH encoding genes affiliated with SAR202 have been found in the hadal zone [33]. However, the lifestyle of SAR202 has only been previously investigated with respect to heterotrophic capabilities, such as amino acid and oligopeptide degradation [34], whilst their potential for CO oxidation has not yet been examined.

In this study, we address the microbial diversity in hydrogen-rich plumes of four Mid Atlantic Ridge (MAR) vent systems: Irinovskoe and Semenov-2 [35], Ashadze-2 [36], and Logatchev-1 [37, 38]. We investigated the ecology and niches of microorganisms inhabiting Irinovskoe and Semenov-2 hydrothermal plumes, and diffuse fluids using long-read 16S rRNA gene analysis, fluorescence in situ hybridization (FISH), and meta-omics approaches. These analyses revealed a niche separation between *Sulfurimonas* and SUP05 in the H_2_-rich plume of Irinovskoe and provide evidence for a potentially carboxydotrophic SAR202 genus.

## Materials and methods

### Site description and sample collection

The four hydrothermal vent fields along the MAR for this study - Irinovskoe, Logatchev-1, Ashadze-2, and Semenov-2, were sampled using the R/V Meteor and the remotely operated vehicle (ROV) Quest (MARUM, Bremen) during expedition “M126”, 18 April – 21 May 2016. Each of these sites has been previously identified as being hosted in ultramafic rock, and either directly measured or suggested to vent H_2_- and CH_4_-rich fluids [35, 37–39], primarily due to subsurface serpentinization reactions. However, the exact concentration of H_2_ varies, as well as the content of other reduced compounds in the fluids (Table 1). Samples were obtained from the endmember fluids for dissolved gas analyses, as well as diffuse fluid above a mussel patch and plume samples for microbiological analyses (Table 2).

**Table 1:** Dissolved gas composition of endmember fluids released at the Irinovskoe, Semenov-2, Ashadze-2 and Logatchev-1 hydrothermal vents.

	**Irinovskoe** ^**a**^	**Semenov-2** ^**a**^	**Ashadze-2** ^**b**^	**Logatchev-1** ^**c**^
**H**_**2**_ **[mM]**	7.5	6.2	26	5.6
**H**_**2**_ **S [mM]**	3.2	4.4	–	2.5
**CH**_**4**_ **[mM]**	0.78	3.5	0.8	1.5
**CO [μM]**	1.9	5.8	–	–

a Estimated endmember uncertainties (2 s) are ±10% (H_2_, H_2_S, CH_4_), ±25% (CO)

b Ashadze-2 chemical composition of fluids is taken from Charlou et al. [38]

c Logatchev-1 chemical composition of fluids is taken from Schmidt et al. [37]

**Table 2:** List of samples from the Irinovskoe (Iri), Ashadze-2 (Ash), Semenov-2 (Sem) and Logatchev-1 (LHF) vent field.

**Name**	**Sample**		**Charact.**	**Device**	**Depth**	**Latitude**	**Longitude**	**δ^3^He (%)**	**∆NTU**	**Potential Temp (°C)**	**Salinity (PSU)**	**DNA extraction**	
**Irinovskoe**													
**Iri_Site1_p**	490CTD_b1		Plume	CTD tow-yo	2616	13.35	−44.90	28.3	0.053	2.73	34.94	P	
**Iri_Site1_bp**	490CTD_b13		Below plume	CTD tow-yo	3282	13.35	−44.90	5.3	0.021	2.38	34.92	P	
**Iri_Site2_rp**	502CTD_b3		Rising plume	CTD vertical	2477	13.33	−44.91	–	0.49	2.85	34.93	P	
**Iri_Site2_p**	502CTD_b5		Plume	CTD vertical	2653	13.33	−44.91	76.7	0.117	2.72	34.93	P	
**Iri_Site2_ap**	502CTD_b19		Above plume	CTD vertical	2568	13.33	−44.91	–	0.011	2.89	34.95	P	
**Iri_Site3_p1**	513CTD_b7		Plume	CTD tow-yo	2600	13.32	−44.91	22.3	0.045	2.75	34.94	M	
**Iri_Site3_p2**	513CTD_b10		Plume	CTD tow-yo	2570	13.34	−44.91	15.8	0.034	2.68	34.93	M	
**Ashadze-2**													
**Ash_Site1_f**	554ROV_2		Fluid	ROV	3277	12.99	−44.91	–	–	15–29^*^	–	P	
**Ash_Site2_rp1**	505CTD_b5		Rising plume I	CTD vertical	3240	12.99	−44.91	127.8	0.132	2.47	34.91	M	
**Ash_Site2_rp2**	505CTD_b12		Rising plume II	CTD vertical	3110	12.99	−44.91	36.6	0.061	2.47	34.92	M	
**Ash_Site2_p**	505CTD_b17		Above plume	CTD vertical	2900	12.99	−44.91	–	0.016	2.52	34.93	M	
**Ash_Site3_rp**	519CTD_b11		Rising plume	CTD vertical	3947	12.97	−44.86	183.1	0.268	2.04	34.88	M	
**Ash_Site3_p**	519CTD_b16		Plume	CTD vertical	3815	12.97	−44.86	14.2	0.058	2.01	34.89	P	
**Semenov-2**													
**Sem_Site1_f**	508ROV_4		Fluid	ROV	2446	13.51	−44.96	–	–	64–135^*^	–	P	
**Sem_Site2_bg**	529CTD_b7		Backgr.	CTD vertical	1880	13.52	−44.96	–	0.01	3.7	35.0	P	
**Sem_Site3_bg**	530CTD_b2		Backgr.	CTD tow-yo	2289	13.52	−44.96	5.2	0.011	2.9	35.0	P	
**Sem_Site4_df**	524CTD_b1		Above mussel field	CTD vertical	2427	13.51	−44.96	266.1	0.027	3.2	34.9	P	
**Logatchev-1**													
**LHF_Site1_bp**	547CTD_b13		Below plume	CTD vertical	2495	14.75	−44.98	11.3	0.014	2.77	34.94	P	
**LHF_Site1_p**	547CTD_b4		Plume	CTD vertical	2727	14.75	−44.98	32.2	0.016	2.59	34.93	P	
**LHF_Site2_df**	544ROV_4		5 cm above mussel bed	ROV	3037	14.75	−44.98	–	–	–	–	P	

DNA extraction protocols: M – DNA Microprep kit and P – PowerSoil DNA isolation kit.

^*^KIPS (Kiel pumping system) collected sample

Plumes were sampled through vertical and tow-yo conductivity, temperature and depth (CTD) casts, using an attached SPE 32 rosette carousel. Diffuse fluids were also sampled from ~1 m above a mussel field at Semenov-2 during a vertical CTD cast. After on-board retrieval of the CTD samples, firstly, a volume of 1 L of seawater was fixed with formaldehyde (1% final concentration) for 10–16 h at 4°C, filtered on 0.22 μm pore size polycarbonate (PC) membrane filters and filters were stored at −20°C for analysis by fluorescence in situ hybridization (FISH). Secondly, a volume of 2 L of seawater was filtered through polyethersulfone (PES) filters, 47 mm diameter and 0.22 μm pore size, and stored at −80°C. Except for the CTD524 samples, in which 30 L of seawater was filtered through 142 mm, 0.2 μm pore size PC filters and subsequently stored at −80°C for downstream ‘omics analysis.

In addition to CTD-based sampling, diffuse fluids were sampled above a mussel bed at Logatchev-1 and Semenov-2, and vent fluids of Semenov-2 and Ashadze-2 were sampled close to the fluid flow, as the fluid emerges from the orifice, using KIPS [37] mounted on ROV Quest. Also, for ROV-based sampling, first, a part of the sample was immediately fixed with formaldehyde (1% final concentration) for 10–16 h at 4°C, filtered on 0.22 μm pore size PC membrane filters and stored at −20°C for FISH. Additionally, an untreated part of the sample was filtered through 0.22 μm pore size PES membrane filters (47 mm diameter, Millipore, Darmstadt, Germany) and preserved at −80°C for downstream ‘omics analysis.

Plume samples were classified based on the turbidity and Eh profile as background water (<0.01 ∆NTU), above or below plume (<0.02 ∆NTU), non-buoyant plume (>0.02 ∆NTU), and rising plume (>0.06 ∆NTU). Samples for helium isotope analysis were also taken and analysed as described in Supplementary Information.

### Endmember fluid measurement

Following the site descriptions in Escartin et al. [35], fluid samples were collected from all high temperature vents at each of the Semenov-2 (Ash Lighthouse, Michelangelo, Yellow Submarine, 311–313°C) and Irinovskoe sites (Pinnacle Ridge, Active Pot, 356–357°C). Samples were collected using isobaric gas-tight (IGT) samplers [40] and processed upon recovery for gas concentrations according to procedures outlined in Reeves et al. [31]. A minimum of two IGT samples were taken from each vent with reported vent temperature above being the maximum stable value measured in real time during fluid collection.

### Thermodynamic calculations

Thermodynamic analysis of Irinovskoe plume was conducted as described previously [9], using the H_2_ and H_2_S concentrations of fluid samples (details: Supplementary Information).

### DNA extraction and sequencing

DNA was extracted from parts of the 142 mm and 47 mm membrane filters using either the ZymoBIOMICS DNA Microprep kit (Freiburg, DE), or the Powersoil DNA isolation kit (MoBio, Ca, USA) according to the manufacturer’s instructions (Table 2). Due to the unavailability of the PowerSoil DNA isolation kit and high demand for DNA kits in autumn 2020, we used the Microprep Kit, as it was the only timely option. Almost full length 16S rRNA genes were amplified using the GM3F and GM4R primer set [41], with barcodes attached to forward and reverse primer. PCR products were pooled in equimolar amounts and sequenced on a Sequel II platform (PacBio, CA, USA) in a circular consensus mode at the Max-Planck-Genome-Centre (Cologne, Germany).

Raw read files were demultiplexed using cutadapt v1.15 [42] and analysed following the DADA2 v1.16.0 (https://benjjneb.github.io/dada2/tutorial.html) [43] pipeline. Alpha diversity was calculated using phyloseq v1.32.0 [44].

### Metagenome and metatranscriptome analysis

DNA extracted from rising plume (Iri_Site2_rp), plume (Iri_Site2_p), and above plume samples (Iri_Site2_ap) of Irinovskoe, along with the diffuse fluid sampled above the mussel field (Sem_Site4_df) of Semenov-2, were used for metagenome analysis (See online supplementary material for a colour version of Table S1). The fragments were sequenced in paired-end mode (2x250 bp length) on a HiSeq2500 platform (Illumina, San Diego, CA, USA) at the Max-Planck-Genome-Centre.

The coverage of the metagenomes was assessed by Nonpareil [45]. Metagenomes were quality trimmed and processed as described previously [9]. In brief, quality trimmed raw reads were assembled using Megahit [46] and Metagenome Assembled-Genomes (MAGs) were retrieved using CONCOCT [47], within Anvi’o v6.0 [48]. Anvi’o was additionally used for visualizing and manually refining MAGs. MAGs were annotated as previously described [9]. Details on MAG analysis are given in Supplementary Information.

Sequence reads from metagenomes of two global expeditions (Malaspina and TARA oceans; Table S2), and those previously published from other hydrothermal plumes, including Macauley on the Kermadec Arc [9], Woody Crack at Menez Gwen at the MAR [34], and from the Lau Basin [6], as well as Von Damm and Piccard [49], and the fluid of Lost City [50] were mapped to MAG contigs using BBMap (99% identity) [51]. This dataset of metagenomes was chosen to better capture and compare the abundances in sulfur-rich and hydrogen-rich plumes (Table S2).

To provide information on the activity of the recovered MAGs in different plume settings, we recruited reads from previously published metatranscriptome datasets from Brothers (Kermadec arc) [9], Piccard (Beebe vents), and Von Damm hydrothermal plumes [49] using BBMap (97% identity) to recovered MAGs. Metatranscriptome analyses were done as previously described [9]. Mapped read values were converted to transcripts per million reads (TPM). The expression of housekeeping genes was estimated by calculating the TPM of several reference genes [52].

### Raw read functional analysis and SNV calculation

Raw reads were annotated and assigned to MAGs using a pipeline described previously [53]. The genes were normalized by the average sequencing depth of 16 universal, single-copy ribosomal genes per sample, following MicrobeCensus v1.1.1 [54]. Genes annotated as [NiFe]-hydrogenase, CODH, and *SoxY*, and their taxonomic affiliations were compared between Irinovskoe samples. We are aware of the limitation of the functional analysis when using short read metagenomes. However, the results of this analysis are used together with other datasets, e.g. cell counts.

Single-nucleotide variations (SNV) were calculated using InStrain [55], following the previously described settings [14].

### Catalyzed reporter deposition-FISH

Catalyzed reporter deposition-FISH (CARD-FISH) analysis followed an established protocol [56]. CARD-FISH details, including probes used in this study, are given in Supplementary Information .

## Results

Samples were taken at four hydrothermal systems between 15–20 fracture zone and marathon fracture zone (12 - 15^0^N) of the MAR: Irinovskoe (Iri), Semenov-2 (Sem), Ashadze-2 (Ash), and Logatchev-1 (LHF). Fluids of these four hydrothermal fields were rich in H_2_, CH_4_, and H_2_S as well as moderately enriched in CO (Table 1). For all four hydrothermal systems, including the novel Semenov-2 and Irinovskoe sites, the vent fluids within each area have been shown to have very similar chemical properties, indicating that each site is fed by a uniform common source fluid [37–39]. For Azhadze-2 and Logatchev-1, endmember gas concentrations reported previously [38] are shown in Table 1. For clarity, sample names are composed of three components: (i) an acronym for the vent (Iri, Ash, Sem, and LHF), (ii) the site which refers to the CTD cast or ROV dive, and (iii) the sample category such as: “f” – hot fluid sample obtained with KIPS directly from the orifice, “rp” - rising plume, “p” - non-buoyant plume, “ap” - above non-buoyant plume, “bp” - below non-buoyant plume, and “df” - diffuse fluid sampled several cm up to 1 m above a mussel bed (Table 2). Two samples, characterized by a very faint or no plume signal were defined as background samples “bg”.

### Microbial community composition

**Figure 1:**
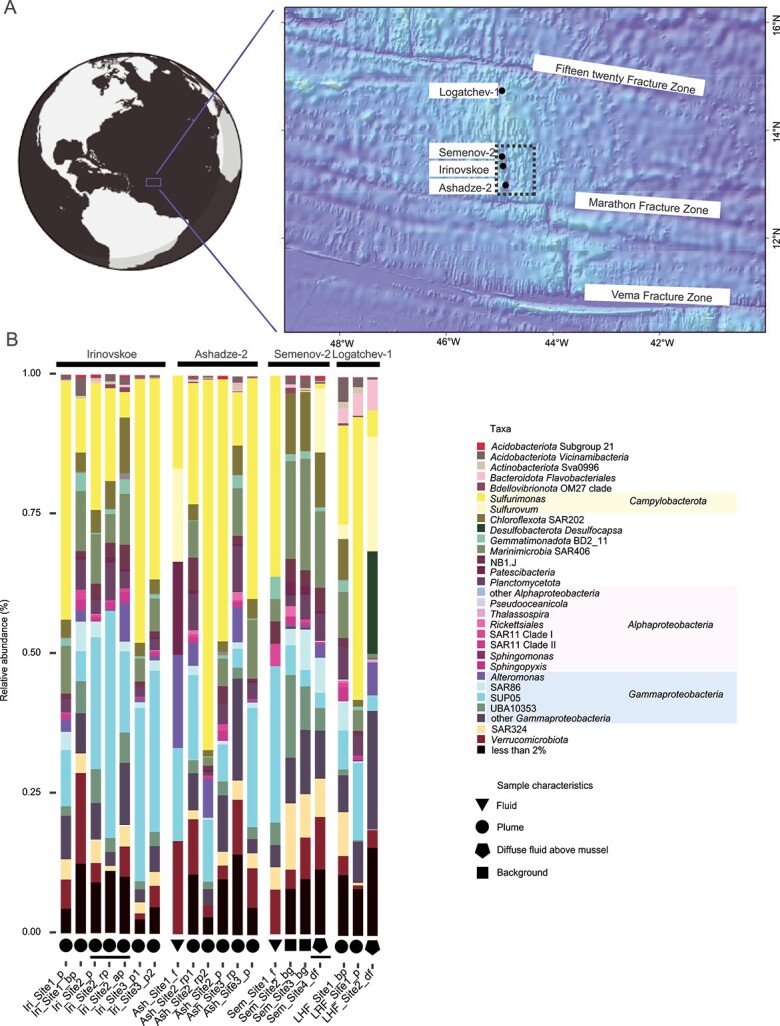
**Relative abundance of 16S rRNA gene amplicon sequence variants (ASVs) in Irinovskoe, Ashadze-2, Semenov-2 and Logatchev-1 samples**. A) Location of four hydrothermal vent fields along the Mid-Atlantic Ridge. B) Full length 16S rRNA gene amplicon sequence variants (ASVs). The barchart depicts the taxonomic assignment of all ASVs annotated at the level of genera with a 2% relative abundance threshold. Samples are grouped based on the location and are given a symbol based on their characteristics: Triangle = fluid, circle = plume, pentagon = diffuse fluid above a mussel field and square = background water. Sample names underscored in black, are those for which metagenome sequences were generated. The ASVs were analysed via a DADA2 pipeline. Sample names consist of: 1) an acronym for the vent (Iri, ash, Sem and LHF), 2) the site which refers to the CTD cast and 3) sample characteristics.

The microbial communities inhabiting different vent fields were compared using full-length 16S rRNA gene amplicons. Amplicon sequencing was performed on samples from hydrothermal fluid (n = 2), diffuse fluid above mussel field (n = 2), hydrothermal plume (n = 14), and background water (n = 2). Although differences were observed in the composition of microbial communities across different sample types and vent fields, several universal features were evident. Plume samples across all four vent fields were rich in *Sulfurimonas* (from 5 to 66%) and SUP05 (from 5% to 40%) (Fig. 1). Additionally, the deep-sea typical clades SAR202 (*Chloroflexota*; from 1% to 10%), SAR324 (from 0.4 to 8%), as well as SAR406 (*Marinimicrobia*; from 1% to 11%) were generally abundant in the plume samples.

Fluid samples of Semenov-2 and Ashadze-2 were also rich in *Sulfurimonas* (up to 36%) and SUP05 (up to 28%). Another abundant clade in the Ashadze-2’s fluid was *Sulfurovum* constituting 17% of the community. In addition, *Sulfurovum* was abundant in the diffuse fluid samples of Semenov-2 (11%) and Logatchev-1 (20%). Based on helium isotopic data, Sem_Site2_bg and Sem_site3_bg exhibited no signal or weak plume signal therefore they were taken as background samples. The community of the background samples was mainly dominated by the typical deep-sea clades, such as SAR202 (11%), SAR324 (12%), and SAR406 (20%). However, in these samples, SUP05 constituted up to 5% of the community, indicating that the plume is influencing the background water in the region. A more in-depth description of the communities of the four vent fields is given in the Supplementary Information.

### Thermodynamic considerations

Thermodynamic calculations revealed that H_2_ and H_2_S oxidation are both thermodynamically favourable in the Irinovskoe plume. As the plume is diluted up to 10^6^-fold (seawater to vent fluid ratio), oxidation of H_2_S releases up to −773 kJ L^−1^, while H_2_ oxidation releases −212 kJ L^−1^ (Table 3). At the low concentrations of CO seen in the pure endmember fluids (1.9–5.8 μM, Table 1), dilution of these concentrations by 10^6^ would have resulted in extremely low (picomolar) concentrations. Hence, CO oxidation energetics were not calculated for the plume.

**Table 3:** Gibb’s free energy available in kJ L^−1^ from the oxidation of H_2_S and H_2_ in the Irinovskoe plume.

**Dilution**	**10^4^**	**10^5^**	**10^6^**
**∆_r_G H_2_**	−222.67	−217.39	−212.12
**∆_r_G H_2_/e^-^**	−111.34	−108.70	−106.06
**∆_r_G H_2_S**	−783.89	−778.61	−773.33
**∆_r_G H_2_S/e^-^**	−97.99	−97.33	−96.67

### Metagenome analysis

Metagenomes were generated for Irinovskoe’s rising plume (Iri_Site2_rp), plume (Iri_Site2_p), and an above plume sample (Iri_Site2_ap), as well as for Semenov-2’s diffuse fluid taken 1 m above a mussel field (Sem_Site4_df). According to Nonpareil [44], the metagenomes covered between 50% and 60% of the microbial diversity of the respective samples (Table S1). To gain further insights into the chemolithoautotrophic processes within the sampled microbial communities, we applied a custom taxonomic and functional annotation pipeline to the metagenomic raw reads [53]. To more accurately compare gene abundances between samples, we determined the number of microbial genomes in each metagenome, using a single-copy marker gene approach via MicrobeCensus v1.1.1 [54], and used this to normalize functional gene counts. With this, we were able to compare the relative abundance and taxonomic affiliation of CO, hydrogen and sulfur oxidation pathways (See online supplementary material for a colour version of Fig. S1). At Irinovskoe, the plume samples contained a higher abundance of the respective oxidation pathway genes compared to the above plume and rising plume. In plume samples, 51% of total genomes contained a small subunit CODH (*coxS*), 38% a [NiFe]-hydrogenase, and 25% a *soxY* gene (See online supplementary material for a colour version of Fig. S1), compared to 49% of genomes with a *coxS*, 26% with a [NiFe]-hydrogenase, and 18% with a *soxY* gene in rising plume and 15% with *coxS*, 5% with a [NiFe]-hydrogenase, and 8% with a s*oxY* gene in above plume. *CoxS* affiliated taxonomically with SAR324, SAR202 (Deep1, Deep2, and other SAR202), *Marinisomatales*, *Gemmatimonadota* (*Longimicrobiales*), *Actinobacteriota* (MedAcidi), *Acidobacteriota* (*Vicinamibacteria*), and *Gammaproteobacteria*. As the hydrothermal fluid is diluted, from the rising plume to the above plume sample, the abundance of genes affiliated with several clades increased, such as SAR202 – Deep2 (up to 5.5% of the total genes), SAR324 (up to 28%), MedAcidi (up to 1.4%), *Marinisomatales* (up to 10.8%), and *Gemmatimonadota* (up to 2.3%). Hydrogenase genes mainly affiliated with SUP05 (48% rising plume, 44% plume, and 33% above plume), *Sulfurimonas* (6% rising plume, 9% plume, and 2% above plume), SAR324 (3% above plume), and other Proteobacteria. Sulfur oxidation genes affiliated with SAR324, *Sulfurimonas*, SUP05, and other *Gammaproteobacteria* and *Alphaproteobacteria*. The highest abundance of sulfur oxidation genes affiliated with SUP05 (55.8%) and *Sulfurimonas* (9.2%) in the plume, while SAR324 genes (56.4%) increased in the above plume sample. Additionally, up to 1% of genomes contained methane monooxygenase genes in the rising and non-buoyant plumes of Irinovskoe.

A total of 47 metagenome-assembled genomes (MAGs) were reconstructed from these metagenomes, with an estimated completeness >40% and contamination <11%. These MAGS were taxonomically affiliated with *Gammaproteobacteria* (SUP05, *Marinobacter, Halomonas, Pseudomonadales, Alteromonas, Idiomarina, Methylococcales, Ketobacter,* and *Alcanivorax*), *Alphaproteobacteria* (*Erythrobacter*), *Campylobacterota* (*Sulfurimonas* and *Sulfurovum*), *Chloroflexota* (SAR202), *Marinosomatales, Acidimicrobia, Planctomycetota, Verrucomicrobiota*, SAR324, and *Poseidoniia* (Table S3). The MAGs reaching the highest relative abundance were those assigned to the SUP05 clade (n = 1), *Campylobacterota* (n = 5), and SAR202 (n = 11). Therefore, these MAGs were not dereplicated, while all other MAGs were dereplicated. Focusing on the dominant MAGs, we performed detailed functional characterizations to predict their metabolic capabilities and ecology.

### Campylobacterota

Five MAGs, affiliated with *Campylobacterota*, were retrieved from Irinovskoe and Semenov-2 metagenomes: MAG_2_2, MAG_17_2, MAG_107, MAG_31, and MAG_104. According to ANI, MAG_104 and MAG_31 belong to the genus *Sulfurovum*, whereas MAG_2_2, MAG_107, and MAG_17_2 belong to the genus *Sulfurimonas* (See online supplementary material for a colour version of Fig. S2 and Fig. S3). MAG_107 and MAG_17_2 shared >95% ANI indicating that they belong to the same species. The same was true for MAG_104 and MAG_31.

**Figure 2:**
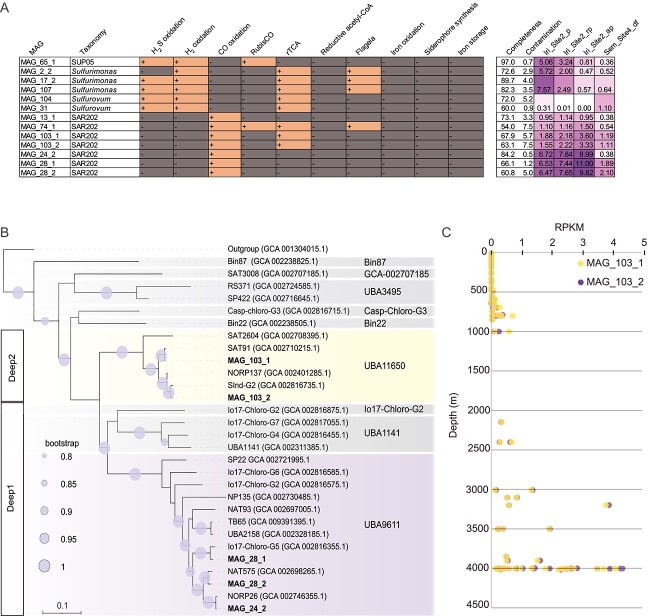
**Metabolic capabilities of retrieved MAGs, phylogenetic analysis of dereplicated SAR202-affiliated MAGs and abundance in the open ocean.** A) Overview of SUP05, *Campylobacterota,* and SAR202 MAGs with a completeness of >60%. On the left hand-side, insights into the metabolic capabilities of the MAGs are given. The plus indicates the presence of the gene or the pathway. The minus indicates the absence of the gene. These MAGs were manually searched for: Sulfur-oxidation genes – Sox genes, H_2_ oxidation – Hydrogenase, CO oxidation – CO dehydrogenase; carbon fixation – RubisCO, rTCA and reductive acetyl-CoA pathway; flagella; iron oxidation – *cyc2* gene, siderophore synthesis and iron storage. On the right hand-side, the estimation of MAG quality and abundance is provided. Abundance is depicted in numbers and purple heatmap. MAGs sharing >95% ANI have one abundance value per metagenome. B) The phylogenetic tree is based on an alignment of 120 bacterial marker genes from SAR202 MAGs included in a) GTDB database [57] and b) Saw et al. [58]. The tree was calculated using GTDB-Tk (https://github.com/Ecogenomics/GtdbTk). C) Abundance of MAG_103_1 and MAG_103_2 in TARA oceans and Malaspina metagenomes spanning the surface water, DCM, mesopelagic and bathypelagic layers.

*Sulfurimonas*-affiliated MAGs dominated the plume samples (up to 7.6 RPKM) (Table S3). The investigation of the metabolic capabilities of these MAGs revealed the presence of both, sulfur and hydrogen oxidation pathways (Fig. 2). Furthermore, *Sulfurimonas* MAGs contained genes for key enzymes of the reverse tricarboxylic acid cycle (rTCA): 2-oxoglutarate:ferredoxin oxidoreductase, pyruvate:ferredoxin oxidoreductase, fumarate reductase, and citrate lyase. These MAGs did not contain genes for iron oxidation, siderophore synthesis or iron storage. In addition, *Sulfurimonas* MAGs contained genes for the formation of flagella (Fig. 2).

*Sulfurovum*-related MAG_31 had a higher abundance in the Semenov-2 diffuse fluid (1.14 RPKM) compared to the plume samples (up to 0.57 RPKM). *Sulfurovum* MAGs also contained genes for rTCA, sulfur, and hydrogen oxidation, but genes for the flagellar machinery were absent.

### SUP05

MAG_65_1 affiliated with SUP05 and shared 93.5% ANI with *Thioglobus vulcanius*, recovered from Brothers plumes [9] (See online supplementary material for a colour version of Fig. S2) and > 95% ANI with Thioglobus_A sp012963715 (See online supplementary material for a colour version of Fig. S4). In contrast to the sulfur-oxidizing *Thioglobus vulcanius*, MAG_65_1 contained not only genes for sulfur oxidation, but also a hydrogen oxidation pathway. This MAG contained the RuBisCO gene for carbon fixation via the CBB pathway. MAG_65_1 was abundant in Irinovskoe, reaching up to 5 RPKM in the plume samples and 3.2 RPKM in the rising plume, whereas in Brothers and Macauley hydrothermal plumes it was up to 1 RPKM (Table S4).

### Phylogenomics of SAR202

SAR202-related MAGs exhibited the highest abundance in the above plume sample in Irinovskoe (up to 11 RPKM) (Fig. 2), a pattern that was also reflected in the ASV analysis (Fig. 1). In order to determine the evolutionary relationships of SAR202 MAGs, phylogenetic trees were reconstructed using previously published MAGs [58] and public MAGs from the GTDB database, as well as the SAR202 MAGs from this study. One tree was built using our high-quality MAGs (Fig. 2) and a second tree using all SAR202 MAGs retrieved here (See online supplementary material for a colour version of Fig. S5). SAR202 has been previously separated into seven distinct groups [59]. Our retrieved MAGs affiliated with three subgroups of the SAR202 clade: Group I, Group II, and Group IIIA (fam. nov. *Ca*. “Monstramariaceae” [59]) (See online supplementary material for a colour version of Fig. S5).

The tree calculation demonstrates that Group IIIA is separated into two different subclades, Deep1 and Deep2. Deep2 formed a distinct genus-level cluster within the family of *Ca*. “Monstramariaceae”, which represents the g_UBA11650 group in the GTDB database. MAGs (n = 4) belonging to this genus have been retrieved from the metagenomes of the TARA oceans mesopelagic layer and surface (SAT2604, SAT91) [60], the North Pond aquifer (NORP137) [61], and the bathypelagic Indian Ocean (SInd-G2) [62].

A 16S rRNA gene tree was built to further inspect the phylogenetic separation of SAR202, using the 16S rRNA genes from: (i) MAG_63_2 (the only recovered MAG with a 16S rRNA gene sequence), (ii) SAR202-related ASVs from Irinovskoe, Semenov-2, and Niua South plume samples [12], and (iii) previously published MAGs (See online supplementary material for a colour version of Fig. S6). SAR202 sequences formed the same groups as the genomic trees, such as Deep2.

Considering all SAR202 MAGs (non-de-replicated dataset), six MAGs belonged to Deep2/UBA11650 in the GTDB database. Among these, MAG_103_1 and MAG_63_1 shared >95% ANI values with each other and MAG SAT91, belonging to the same species. In addition, MAG_103_2 and MAG_63_2 shared >95% ANI with each other and SInd-G2. Two MAGs assigned to Deep2 and with the lowest contamination (MAG_103_1 and MAG_103_2) were further investigated regarding their metabolism.

### Functional capabilities of SAR202 Deep2

A functional analysis of MAG_103_1 and MAG_103_2 revealed the presence of all key genes of the rTCA. SAR202 MAGs did not harbour genes for sulfur and hydrogen oxidation but contained genes encoding the formate dehydrogenase for formate oxidation. Genes coding for proteins of the taurine, hypotaurine, methanosulfonate, and dimethylsulfoniopropionate degradation pathways have been previously reported for deep-sea SAR202 [62]. However, these genes were missing in the MAGs recovered here and the previously published, more complete MAGs (e.g. SAT91).

**Figure 3:**
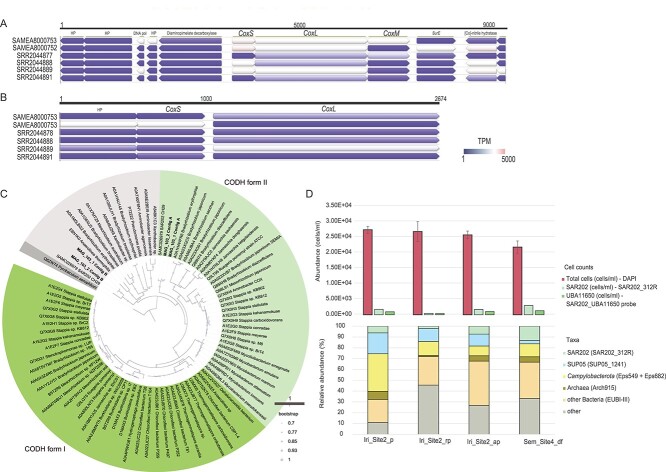
**Arrangement of the complete CODH operon and the partial CODH operon in MAG_103_1, phylogenetic analysis of CODH and cell counts and relative abundance of selected microbial taxa.** A) a complete CODH operon in MAG_103_1 and B) a partial CODH operon in MAG_103_1. Metatranscriptomes of brothers (SAMEA8000753, SAMEA8000752), Von Damm (SRR2044878, SRR2044888) and Beebe (SRR2044889, SRR2044891) plumes were mapped to MAG_103_1 with a minimum identity of 97%. The transcription is denoted in several lanes of arrows beneath the genes and their colour indicates the transcript per million (TPM). Visualization was done with Geneious 11.1.5. C) Phylogenetic tree of large subunit CODH (*coxL*) using SAR202-affiliated MAGs and 78 published sequences. Tree was calculated with FastTree [64]. D) The upper part depicts the total cell counts determined by counting DAPI stained cells and cells targeted by probe SAR202_312R probe, as well as the specific probe SAR202_UBA11650, which targets the previously uncharacterized SAR202 genus, provisionally named “UBA11650” in the GTDB database. On the lower part of the plot the abundance of the microbial groups relative to the number of DAPI stained cells is given.

Both SAR202 MAGS harboured two putative CODH operons. The large subunit *coxL* contained the AYRGAGR motif [63], indicating that these operons encode the form II CODHs. This was also supported by the operon structure (S-L-M; Fig. 3). The second operon detected in both MAGs contained only L-S subunits, potentially due to incompleteness of the contigs. Other closely related MAGs (SAT91 and Sind-G2) contained two complete operons.

A phylogenetic tree using the *coxL* gene of MAG_103_1, MAG_103_2, and 78 published sequences was calculated (Fig. 3). This analysis revealed that the *coxL* of the complete operon (contig A) indeed affiliates to CODH form II. In addition, *coxL* from MAG_103_1 and MAG_103_2 were most closely related to those of SAR202 CH29 recovered from the hadal zone [33]. The *coxL* of the partial operons (subunits L-S) affiliated with the putative sequences belonging to molybdenum hydroxylases family.

### Transcribed metabolic pathways

To investigate the functional niches of the dominant microbes identified here, we used previously published metatranscriptomes from vent fields with contrasting and similar chemical properties. Metatranscriptomes were chosen in order to represent hydrogen-poor (Brothers plume [9]) and hydrogen-rich plumes (Von Damm and Piccard - Beebe vent [49]). Metatranscriptomes from hydrogen-rich plumes share similar chemical properties with Irinovskoe (Table 1), e.g. Von Damm (H₂ - 9.9-19.2 mM; H₂S - 3.15 mM) [16] and Beebe (H₂ - 19.9 mM; H₂S - 22 mM).

*Sulfurimonas* and SUP05 transcription was investigated in four Von Damm metatranscriptomes, two from rising plume and two from neutrally-buoyant plume [49] (See online supplementary material for a colour version of Fig. S7). The second plume sample (SRR2044888) is further from the seabed and could represent a more diluted plume sample. The analysis revealed a higher transcription of *Sulfurimonas*-related small subunit [NiFe]-hydrogenase in the plume sample (42 293 TPM), compared to the rising plume (12 514 TPM) (See online supplementary material for a colour version of Fig. S7). The transcription of the large subunit decreased slightly between rising plume (12 500 TPM) and plume (10 158 TPM). SUP05, in contrast, exhibited a high transcription of hydrogenases in the rising plume (282 591 TPM). As the plume diluted, the hydrogenase transcription decreased (61 619 TPM), whereas the Sulfide:quinone oxidoreductase (s*qr*) gene, indicative of sulfur oxidation, became more transcribed, reaching 135 169 TPM (See online supplementary material for a colour version of Fig. S7).

MAG_103_1 was highly transcribed in Brothers (housekeeping gene average = 1349 TPM), Von Damm (housekeeping gene average = 728 TPM), and Beebe (housekeeping gene average = 258 TPM). The transcription of four key genes of the rTCA pathway and CODH in MAG_103_1 was higher than that of the housekeeping genes. Transcription of Pyruvate:ferredoxin oxidoreductase gene (rTCA) of MAG_103_1 reached up to 1199 TPM in Brothers, 1273 TPM in Von Damm, and 1141 TPM in the plume of Beebe. CODH was also highly transcribed in Brothers (4619 TPM), Von Damm (1271 TPM), and Beebe (731 TPM).

MAG_103_2 exhibited a high transcription in Von Damm (2563 TPM - Pyruvate:ferredoxin oxidoreductase; 3310 TPM - CODH) and Beebe (1710 TPM - Pyruvate:ferredoxin oxidoreductase; 3366 TPM - CODH). CODH was not highly transcribed in Brothers plume (210 TPM - CODH) (See online supplementary material for a colour version of Fig. S8).

### Visualization and quantification of cells

CARD-FISH was conducted on the four samples for which metagenomes were sequenced, to confirm the relative abundance of specific clades, such as SUP05, *Campylobacterota*, and SAR202 (Fig. 3). The total cell counts were in the range of 2.32 × 10^4^ to 2.90 × 10^4^ cells ml^−1^ in Irinovskoe plume and 2.21 × 10^4^ cells ml^- 1^ in Semenov-2’s diffuse fluid. Bacteria dominated the community in both hydrothermal systems (Irinovskoe: 81%; Semenov-2: 62%) over Archaea (Irinovskoe: 7%; Semenov-2: 5%). SUP05 reached up to 19% of the community in plumes of Irinovskoe, and only 3% in the diffuse fluid of Semenov-2. In agreement with 16S rRNA amplicon analysis, SAR202 was most abundant in diffuse fluid of Semenov-2, at 13%, compared with 6% in Iri_Site2_p, 2% in Iri_Site2_rp, and 7% in Iri_Site2_ap.

CARD-FISH confirmed the high abundance of *Campylobacterota* reaching 35% in Irinovskoe and 12% in Semenov-2. In Irinovskoe samples, *Campylobacterota* was only visualized as free-living bacteria, whereas in Semenov-2 *Campylobacterota* formed long bacterial chains.

A probe was designed to target the SAR202 clade UBA11650 (Deep2). The genus constituted 4% of the community in the Irinovskoe’s above plume sample (1.0 × 10^3^ cell ml^- 1^) and 6% (1.3 × 10^3^ cell ml^− 1^) in Semenov-2. Cells were coccoid with a diameter of 1–1.5 μm (Fig. S9).

### Biogeography and evolution of SAR202 Deep1 and Deep2

The abundance of the SAR202-affiliated, MAG_103_1 and MAG_103_2 (Deep2/UBA11650) was investigated in metagenomes from plumes (Lau Basin, Macauley, Irinovskoe, Von Damm, and Beebe), diffuse fluids (Piccard, Von Damm, and Semenov-2), as well as fluids (Lost City) (Table S2). MAGs belonging to Deep2 retrieved in this study were highly abundant in plumes of Irinovskoe (3.6 RPKM), Beebe (0.9 RPKM), and the diffuse fluid of Semenov-2 (1.19 RPKM). A lower abundance (0.4 RPKM) was observed in Mariner plumes. Additionally, only a few reads of Macauley, Menez Gwen, and Tui Malila metagenomes were recruited to these MAGs, indicating the absence of them in the aforementioned plumes.

MAG_28_2 (Deep1) abundance was also compared to that of Deep2 MAGs (Table S5). This MAG was abundant in the Irinovskoe plume (9.8 RPKM) and Semenov-2 diffuse fluid (2.1 RPKM), however it was not detected in high abundance in other metagenomes (See online supplementary material for a colour version of Fig. S10).

To assess the abundance of Deep2 in the open-ocean, the metagenomes of Malaspina expedition and TARA Oceans were recruited (Table S2, Table S5). Deep2 MAGs exhibited a low abundance in TARA Oceans metagenomes and reached >4 RPKM in the deep-sea (3000–4000 m depth) (Fig. 2).

## Discussion

Hydrothermal fluids released from ultramafic-hosted vent fields along tectonic plates represent a significant input of energy and reduced chemical compounds to the ocean that influence microbial communities of hydrothermal plumes and the wider deep sea. Here, we analysed the communities of four hydrogen-rich hydrothermal systems along the MAR (Irinovskoe, Semenov-2, Logatchev-1, and Ashadze-2), and conducted an in-depth analysis on the ecology and niche partitioning of *Sulfurimonas*, SUP05, and SAR202. Here, we report that some members of the SAR202 clade contain and transcribe the genes for the CO oxidation (CODH gene) and rTCA, indicative of chemolithoautotrophic growth.

### Sulfur and hydrogen oxidation in MAR plumes

*Sulfurimonas* and SUP05 clade dominated the plume communities of the four vent fields investigated in this study. *Sulfurimonas* represented up to 66% relative abundance of plume communities, supporting a previous report of their dominance in the Logatchev vent field plume [65]. Both *Sulfurimonas* and SUP05 are reported to be abundant in hydrogen-rich plumes [66, 67], however, the mechanisms controlling their niche separation from rising to the non-buoyant plume are not as of yet clear.

Here, we identified an increase in the *Sulfurimonas*-affiliated [NiFe]-hydrogenase gene from the rising plume to the non-buoyant plume (See online supplementary material for a colour version of Fig. S1). Similarly, based on four metatranscriptomes of another hydrogen-rich plume (Von Damm) [49], the transcription of the small subunit of [NiFe]-hydrogenase increased from rising plume to plume and decreased in the furthest sample from the seabed (See online supplementary material for a colour version of Fig. S7), whereas the transcription of the large subunit exhibited a small decrease (12 000 TPM – 10 000 TPM). A higher expression of the small subunit compared to the large subunit has also been previously reported for cultivated *Sulfurimonas denitrificans* [68], using proteomics. Together, the increase in hydrogenase gene number and transcription indicated that *Sulfurimonas* could be gaining energy through H_2_ oxidation as the plume becomes more diluted.

Although *Sulfurimonas* is potentially gaining energy by oxidizing H_2_, we estimate that the energy yield from the aerobic oxidation of H_2_S (−773.33 kJ L^−1^) would be considerably higher than that of H_2_ (−212.12 kJ L^−1^) at a 1:10^6^ fluid dilution in Irinovskoe plume samples (Table 3). However, due to the high negative redox-potential, hydrogen likely reduces the NAD(P)/H without the need of a reverse electron chain [7]. Therefore, the energy required for fixing 1 mol C when oxidizing hydrogen (1060 kJ) is one third of that needed when oxidizing sulfide (3500 kJ) [69]. Additionally, while *Sulfurimonas* species are known to encode up to six Sqr genes [70], the three *Sulfurimonas* MAGs recovered in this study harbor only one type, Sqr VI. Studies of Sqr Type VI in *C. tepidium* have shown that it is specialized to function at high sulfide concentrations (>4 mM) and thus could be utilized by *Sulfurimonas* only near fluid release sites [70]. This supports the theory that *Sulfurimonas* can likely reach and sustain large population sizes in diluted, non-buoyant plumes by performing H_2_ oxidation.

The SUP05 MAG retrieved from the plume of Irinovskoe shared >95% ANI with Thioglobus_A sp012963715 (See online supplementary material for a colour version of Fig. S4), thus, a combination of phylogenetic analysis and functional annotation allowed us to propose a new name for this species, “*Thioglobus ultramaficus”*. *T. ultramaficus* harboured genes for a complete hydrogen oxidation pathway (Fig. 2), similar to previously recovered MAGs of hydrogenotrophic SUP05 from the Guaymas Basin plumes [2]. In contrast to *Sulfurimonas*, however, we observed a decrease in the transcription of hydrogen oxidation genes and an increase in the transcription of the sulfur oxidation gene as the plume dilutes, based on the Von Damm’s metatranscriptome. This observation suggests a transition in the functional niche of SUP05 as the plume becomes more diluted, from hydrogen to reduced sulfur compounds, potentially due to high affinity for sulfur that SUP05 species have exhibited [71]. In fact, *T. ultramaficus* contained two Sqr gene, type VI and IV, enabling this species to cope with varying sulfide concentrations [70]. Although closely related to SUP05 species inhabiting the metal- and sulfur-rich plumes of Brothers and Macauley in the Kermadec Arc, *T. ultramaficus* appeared to be less abundant in those plumes (up to 1 RPKM), likely due to differences in plume chemistry. Although high iron concentration has been shown to influence SUP05 abundance, as reported through statistical analysis [9] and Fe-amended incubation [72], the selective conditions of hydrogen in the rising plume and sulfur in non-buoyant plume seem to favour *T. ultramaficus* over other SUP05 species. Furthermore, this corroborates findings in the non-buoyant plume of Polaris (Gakkel Ridge), where SUP05 (up to 47% abundance) has been reported to rely on sulfur oxidation; in contrast, hydrogenase transcripts were assigned mainly to *Sulfurimonas* (up to 19% abundance) [67].

**Figure 4:**
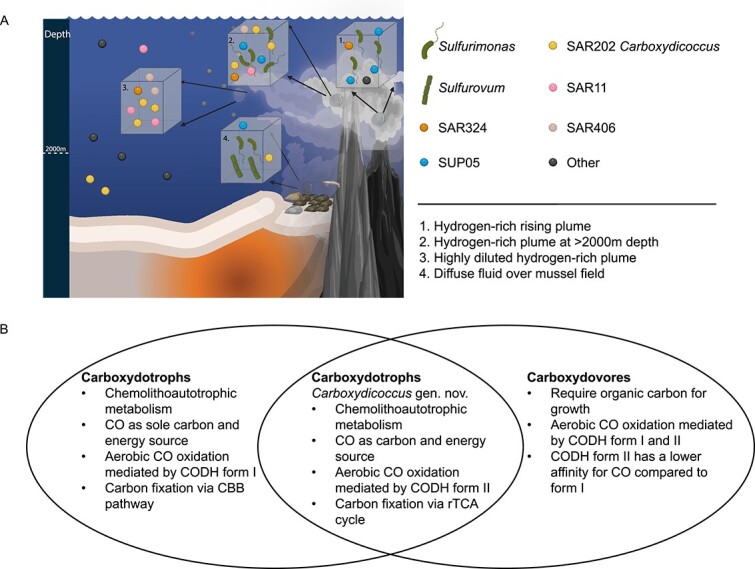
**Overview of the hydrogen-rich plumes at the Mid-Atlantic Ridge and the metabolic capabilities of the new SAR202 genus.** A) Sketch depiction of the communities in different stages of the plume in the hydrogen-rich plume such as: The rising plume, non-buoyant plume, highly diluted plume and diffuse fluid. Created with BioRender.com. B) Comparison between the caboxydotrophic and carboxydovoric microorganisms and the features of the new SAR202 genus.

Through the investigation of community composition and functional roles in the rising and non-buoyant plume samples, we identified a niche partitioning between *Sulfurimonas* and SUP05 based on hydrogen and sulfur oxidation (Fig. 4). It appears that *Sulfurimonas* persistently made use of hydrogen in both plume stages, while SUP05 transitioned from hydrogen to sulfur oxidation from the rising to the non-buoyant plume.

### Potential chemolithoautotrophy in the mixotrophic clade SAR202 Deep2

Among the abundant clades in MAR plumes is the SAR202 deep-sea clade (Fig. 1). SAR202 has previously been reported to reach up to 3–4% in the deep-sea [58], whilst it constituted up to 15% of the community in hydrothermal plumes of Woody Crack in MAR [34]. Similarly, in the plumes of Irinovskoe, Semenov-2, Ashadze-2, and Logatchev-1 vent fields investigated here, the SAR202 accounted for up to 13% of the microbial community (Fig. 1, Fig. 3). SAR202-affiliated MAGs retrieved from Irinovskoe and Semenov-2 belonged to Group I, II, and Group IIIA – Deep1 and Deep2 clades (Fig. 2). An in-depth investigation of Deep2 revealed that several SAR202 MAGs were assigned to a genus-level cluster, provisionally named “UBA11650” in the GTDB database. By combining phylogenetic analysis, detailed functional annotations, visualization, and enumeration of environmental cells, we were able to provide enough information for members of the “UBA11650” cluster, to propose a novel genus within the *Ca.* Monstramariaceae family, termed “*Carboxydicoccus”* (Table S6; described under SeqCode). MAGs belonging to this genus have been retrieved from Iri_Site2_rp (2 MAGs), Sem_Site4_df (2 MAGs), as well as from diffuse fluid of the North Pond (Mid-Atlantic Ridge), and the mesopelagic layer of South Atlantic and Indian oceans [61, 62]. We propose that the type of this new genus is “*Carboxydicoccus profundi”*, with the type material being the previously published MAG SAT91 [60], due to its high completeness. The naming refers to the ability of this species to oxidize CO in the deep sea and its coccoid morphology (See online supplementary material for a colour version of Fig. S9). SAT91 was recovered from a co-assembly of South Atlantic TARA metagenomes and reaches a high abundance in TARA mesopelagic samples (up to 0.7 RPKM).

*Carboxydicoccus* is abundant in hydrothermal plumes and the deep sea. Using a novel, genus-specific probe (SAR202_UBA11650), we found that *Carboxydicoccus* reached up to 4% relative abundance in hydrothermal vent plumes of Irinovskoe (1.0 x 10^3^ cells ml^- 1^) and up to 6% in the diffuse fluid of Semenov-2, (1.3 x 10^3^ cell ml^- 1^). This FISH based quantification is well in line with 16S rRNA gene analysis. The genus specific probe SAR202-UBA11650 will in the future facilitate the identification and quantification of the CO utilizing bacteria of the novel genus *Carboxydicoccus* in plume samples.

The analysis of functional gene content and transcription of *Carboxydicoccus* MAGs in hydrothermal vent field samples indicates a chemolithoautotrophic lifestyle through CO oxidation and carbon fixation (rTCA). In oxygenated hydrothermal plumes, CO derived from endmember fluids, reaching up to 7.4 μM in most H_2_-rich bare rock systems [16], is not expected to be available in high concentration (low pM) due to extensive dilution (>10^4^ fold [1]). CO concentrations are generally maintained at low μM concentrations in H_2_-rich hydrothermal fluids by relatively rapid thermodynamic equilibrium with dissolved CO_2_ and H_2_ (termed the “water-gas shift” reaction), a reaction generally invariant over large temperature changes [15]. CO concentrations from Semenov-2 and Irinovskoe presented here (Table 1) are within the range given above and therefore, consistent with equilibrium according to this reaction. This implies that, unlike other reduced carbon species, such as formate, no additional abiotic CO production can be expected during dilution and mixing of the hydrothermal fluids presented, based on both thermodynamic and experimental considerations [15]. Previous studies have, nonetheless, suggested increased plume CO/CH_4_ ratios as the plume gets further diluted due to microbial activity [73]. In that study, the authors proposed that partial decomposition of CH_3_OH formed during microbial CH_4_ oxidation could result in microbial CO production in the plume, thus increasing concentrations in the non-buoyant plume relative to the exceedingly low abiotic CO in the source fluids. Given that the oxidation of CO is likely faster than CH_4_ [73], this would imply an active source to maintain elevated CO/CH_4_ ratios. It is therefore possible that the CO oxidation suggested in the genes studied here is fuelled indirectly by incomplete microbial oxidation of the copious CH_4_ (Table 1) in the fluids. In fact, genes for methane oxidation were found in the rising and non-buoyant plume, specifically in a Methylococcales MAG.

Genes for the oxidation of CO were present in a higher number in the rising plume and plume than in the above plume genomes (See online supplementary material for a colour version of Fig. S1). The decrease in CO oxidation capability in the above plume community likely reflects the lower concentration of CO in surrounding seawater. However, as the total CO oxidation genes decreased in number, the proportion of the *Carboxydicoccus* -affiliated CODH genes increased. Additionally, we observed a high transcription of CO oxidation genes and those of the rTCA pathway by *Carboxydicoccus* in two hydrogen-rich non-buoyant plumes (Von Damm and Beebe; [49]) and one metal-rich plume (Brothers) (See online supplementary material for a colour version of Fig. S8). The high transcription of these genes indicates that CO could be serving as carbon and energy source.

In addition, SNV analysis (Table S7) revealed that the population of *Carboxydicoccus* is more diverse in the Irinovskoe plume compared to the deep sea and their genes are under diversifying selection. This form of natural selection favours the extreme traits and drives speciation. Here, we conclude that *Carboxydicoccus* thrives in plume communities and it persists in the low-carbon deep sea.

### CODH form II

Two copies of CODH operons were found in each SAR202 MAG of *Carboxydicoccus*, one complete and one incomplete. *CoxL* contained the AYRGAGR motif and, together with other subunits, were organised in the order of small-large-medium, indicating that they belonged to CODH form II. Such proteins have previously been reported to have a lower affinity compared to form I and still have a putative function [74].

It has been reported that potentially the primary function of CODH form II is not CO oxidation [74]. This conclusion was reached since cultivated *Roseobacter* species that contained only CODH form II lacked the accessory genes. The importance of accessory proteins in CODH proteins has been observed when mutagenic disruption of these genes impaired the ability to synthesize a functional CODH [75, 76]. In contrast to CODH form I, form II does not have a consensus order of the accessory **c*ox* genes. In fact, these genes have been found in other parts of the *Carboxydicoccus* genomes. This arrangement could make it easier for bacteria to lose these necessary genes, which could have been the case for *Roseobacter* species. However, in MAG_103_1 and MAG_103_2, the accessory genes including *coxF*, *coxE*, and *coxD* were present and transcribed (See online supplementary material for a colour version of Fig. S11).

CODH form II have been predominantly found in carboxydovoric microorganisms, as described in King [26], and King and Weber [19]. Such microorganisms use the ability to oxidize CO as supplementary energy source [77]. However, SAR202 MAGs contain a complete rTCA cycle, thus potentially harbouring a carboxydotrophic metabolism. Several cultivated carboxydotrophic organisms have been reported to utilize CO oxidation for energy and carbon assimilation through CBB cycle [26, 28, 74]. However, carboxydotrophic bacteria using rTCA have not been described yet (Fig. 4). An *in silico* comparison of the expense using CBB and rTCA, revealed that biomass production from CO_2_ is less energetically expensive using the rTCA compared with the CBB cycle [78]. Nevertheless, a clear comparison of the growth yield driven by diverse carbon fixation pathways is complicated since several variables need to be taken into account such as oxygen concentration and the electron chain of different bacteria. The ability of *Carboxydicoccus profundi* to oxidize CO and fix carbon in hydrothermal plumes needs to be further investigated via cultivation experiments. Based on phylogenetic analysis, detailed functional annotations, and visualization, we hypothesize that *C. profundi* relies in its energy and carbon requirement on the chemolithoautotrophic utilization of the low CO concentration in plumes. Carboxydotrophy thereby becomes a niche defining trait of *C. profundi* in plumes and substrate-deprived bathypelagic habitats.

## Supplementary Material

Dede_et_al_Supplementary_Figures_Round1_wrae165

Supplementary_Information_BD_wrae165

## Data Availability

Metagenomes and MAGs were submitted to the European Nucleotide Archive and are available under project PRJEB60262. 16S rRNA gene reads are available under PRJNA1090079.
