# Supplementary material for: Bacterial chemolithoautotrophy in ultramafic plumes along the Mid-Atlantic Ridge
Source: ISME J. 2024 Aug 20;18(1):wrae165. doi: 10.1093/ismejo/wrae165 (PMC11411561; doi:10.1093/ismejo/wrae165)
Supplement: Dede_et_al_Supplementary_Figures_Round1_wrae165 [file dede_et_al_supplementary_figures_round1_wrae165.pdf]

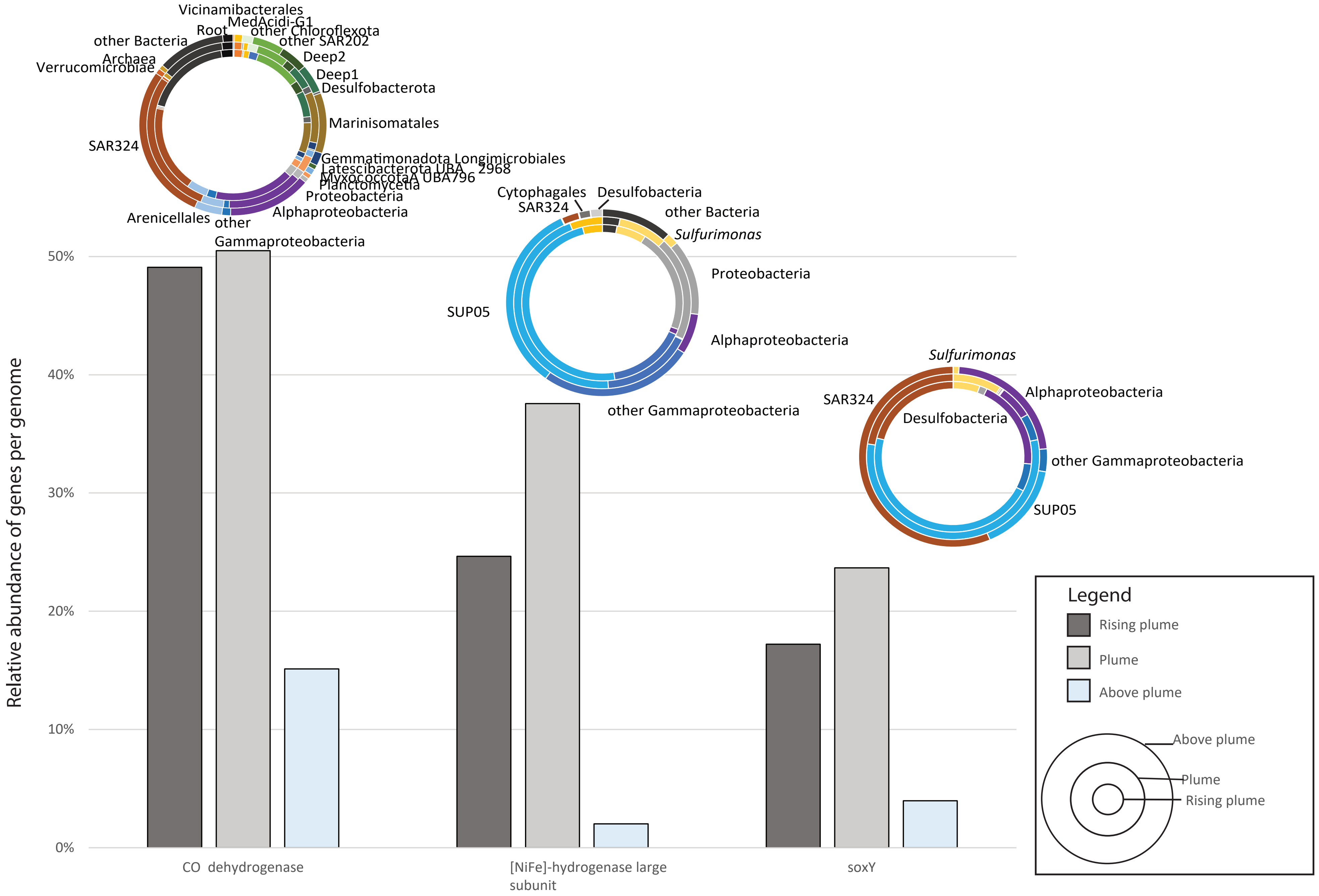

Figure S1: Relative abundance of CODH small subunit, [NiFe]-hydrogenase large subunit and *soxY* gene per genome depicted in barchart. These three genes were analysed in the rising plume (Iri_Site2_rp), plume (Iri_Site2_plume) and above plume (Iri_Site2_ap). The pie charts depict the taxonomic affiliation of these three genes. The taxonomic relative abundance of each gene, given in pie charts, is located above the bar representing the abundance of that gene per genome. The inner most circle refers to the rising plume, the middle circle refers to the plume and the outer circle refers to above plume sample.

A)

	MAG_104	MAG_31	*Sulfurovum* sp. FS06-10	MAG_17_2	MAG_107	MAG_2_2	*Sulfurimonas* UBA2011	*Sulfurimonas autotrophica*	*Sulfurimonas hongkongensis*	*Sulfurimonas denitrificans*	Bin_42	Sulfurimonas_3	Sulfurimonas_4	Sulfurimonas_5	Sulfurimonas_6	Sulfurimonas_7
MAG_104	*															
MAG_31	96.6	*														
Sulfurovum FS06-10 (GCA_000830185.1)	72.9	73.4	*													
MAG_17_2	66.7	67.0	66.3	*												
MAG_107	66.5	67.0	67.0	99.1	*											
MAG_2_2	66.5	66.4	66.4	88.9	89.1	*										
S. UBA2011 (GCA_002335055.1)	66.6	66.6	66.6	90.0	90.3	98.8	*									
S. autotrophica (*GCA_011322265.1*)	66.2	67.1	67.1	72.9	72.8	72.7	72.7	*								
S. hongkongensis (GCF_000445475.1)	66.1	66.8	66.8	71.7	71.8	71.8	71.7	71.0	*							
S. denitrificans (GCF_000012965.1)	65.7	67.0	67.0	71.2	71.3	71.2	71.0	71.1	73.3	*						
Bin_42 Kermadec (GCA_910596195.1)	66.4	66.9	66.9	88.8	88.8	88.4	88.5	73.1	71.8	70.9	*					
Sulfurimonas_3 (GCA_900117435.1)	67.2	67.6	67.3	72.4	72.4	72.2	72.2	72.3	71.7	71.7	72.2	*				
Sulfurimonas_4 (GCA_900118305.1)	66.1	66.5	66.5	87.9	88.2	91.9	91.8	72.1	71.0	70.0	87.8	70.7	*			
Sulfurimonas_5 (GCA_900118315.1)	67.2	67.8	67.8	73.0	73.1	73.3	73.1	75.2	71.3	71.7	73.0	73.1	72.2	*		
Sulfurimonas_6 (GCA_900118345.1)	66.7	66.9	66.9	72.2	72.2	72.2	72.1	75.0	70.8	70.7	72.5	72.0	71.4	76.1	*	
Sulfurimonas_7 (GCA_900118175.1)	66.2	65.8	65.8	69.1	69.0	69.3	69.1	69.5	69.2	69.0	69.0	73.0	68.5	70.7	70.7	*

B)

	MAG_65_1	*Ca.* T. vulcanius	SUP05 GB-1	SUP05 GB-2	Ca. T. autotrophicus	*Ca.* T. thermophilus	*Ca .* T. vadi	*Ca.* T. plumae	*Ca.* T. singularis	SUP05-7_Brothers
MAG_65_1	*									
Ca. T. vulcanius (GCA_910595855.1)	93.8	*								
SUP05 GB-1	78.8	74.2	*							
SUP05 GB-2	74.1	74.0	82.0	*						
Ca. T. autotrophicus (GCA_001293165.1)	77.1	77.3	74.0	73.7	*					
Ca. T. thermophilus (GCF_001875585.1)	72.4	72.1	73.8	74.0	72.7	*				
Ca . T. vadi (GCA_910595835.1)	74.3	73.9	79.0	77.1	73.8	73.4	*			
Ca. T. plumae (GCA_910595815.1)	74.3	74.4	73.9	80.8	73.9	74.1	81.1	*		
Ca. T. singularis (GCA_003326015.1)	67.6	67.5	67.8	67.7	67.3	67.6	67.5	67.7	*	
SUP05-7_Brothers (GCA_910595995.1)	68.6	68.6	68.5	68.4	68.5	68.4	68.8	68.5	74.7	*

Figure S2: Average nucleotide identity (ANI) between MAGs retrieved in this study and published genomes. A) *Sulfurimonas* and *Sulfurovum* MAGs retrieved in this study were compared to genomes of cultivated species and *Sulfurimonas* MAGs (Sulfurimonas_1 to Sulfurimonas_7) retrieved from the Manus Basin. B) Free-living SUP05 MAGs retrieved in this study and published genomes. Threshold; genus >70%; species >95%, labelled with light and dark brown, respectively.

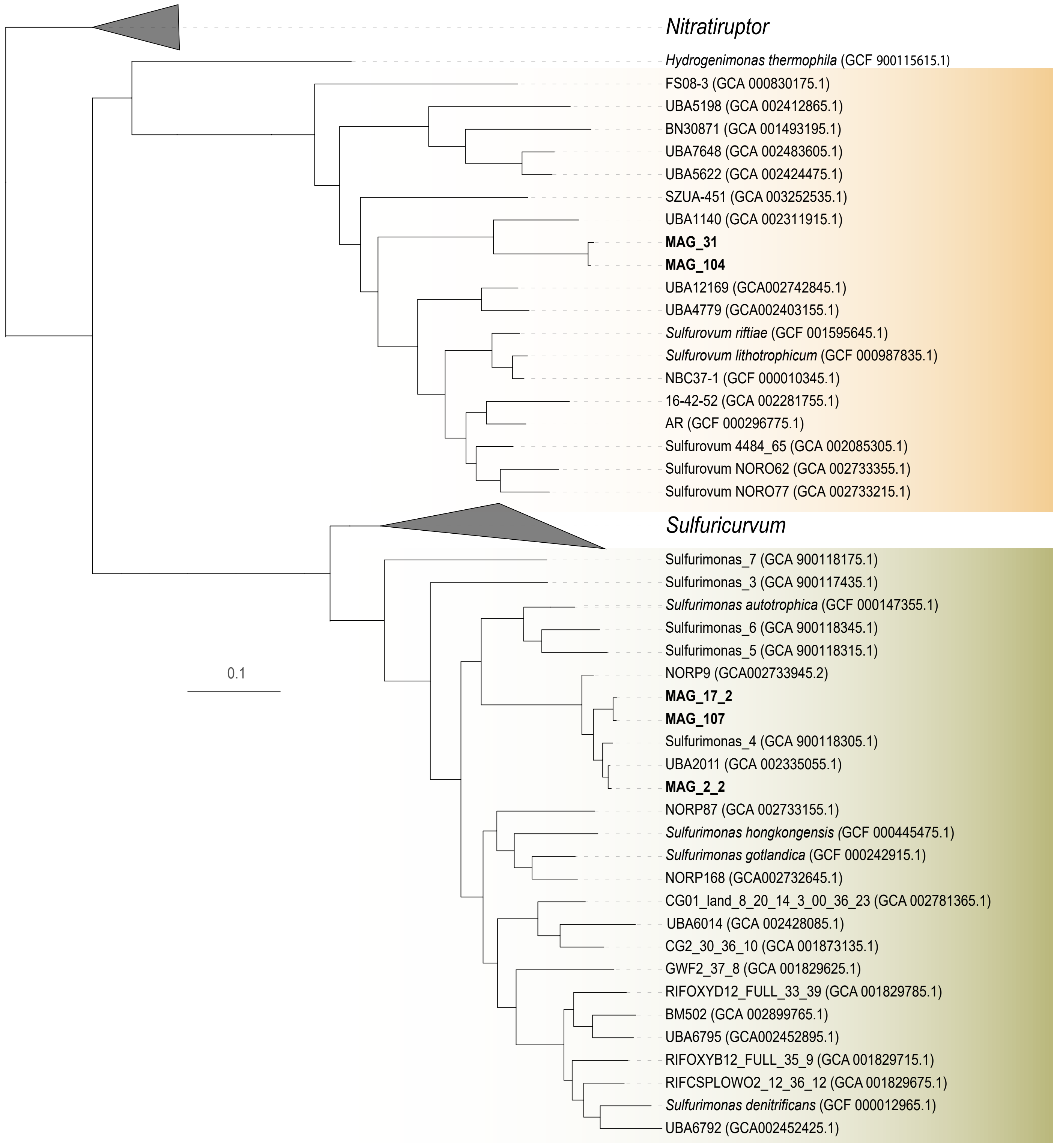

Figure S3: Phylogenetic analysis of *Sulfurimonas* genomes. The phylogenetic tree is based on an alignment of 120 bacterial marker genes from published Sulfurimonas MAGs in the GTDB database and MAGs obtained in this study. The tree was calculated using GTDB-tk.

A)

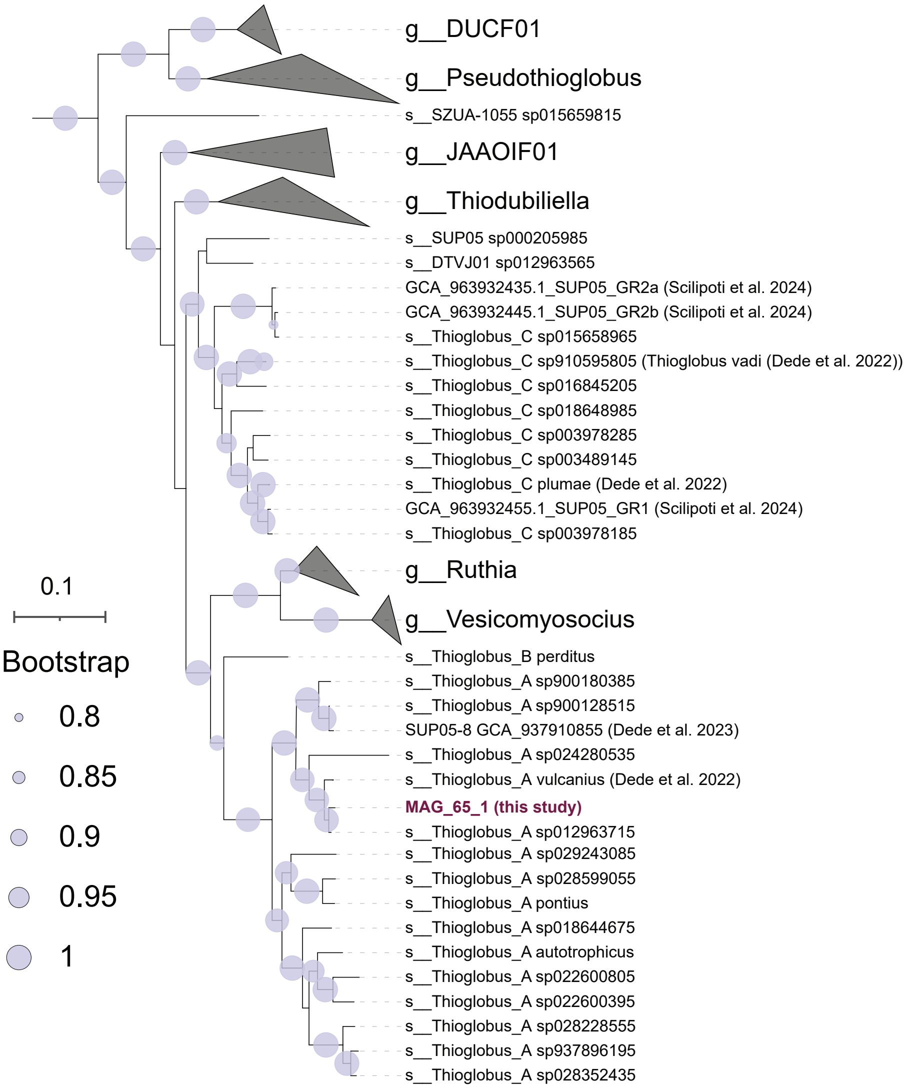

B)

		Thioglobus_A sp012963715		
	MAG_65_1	GCA_012964205.1	GCA_029938145.1	Thioglobus vulcanius
MAG_65_1	*	96.56	95.09	93.86
GCA_012964205.1	95.62	*	95.8	93.99
GCA_029938145.1	95.04	96.78	*	95.56
Thioglobus vulcanius	93.76	94.72	95.54	*

Figure S4. Phylogenetic analysis and ANI of SUP05 genomes. A) The phylogenetic tree is based on an alignment of 120 bacterial marker genes from published SUP05 MAGs in Scilipoti et al. (34), the GTDB database (r220) and MAGs obtained in this study. The tree was calculated using GTDB-tk. B) ANI values of MAG_65_1 and its closely related species.

A)

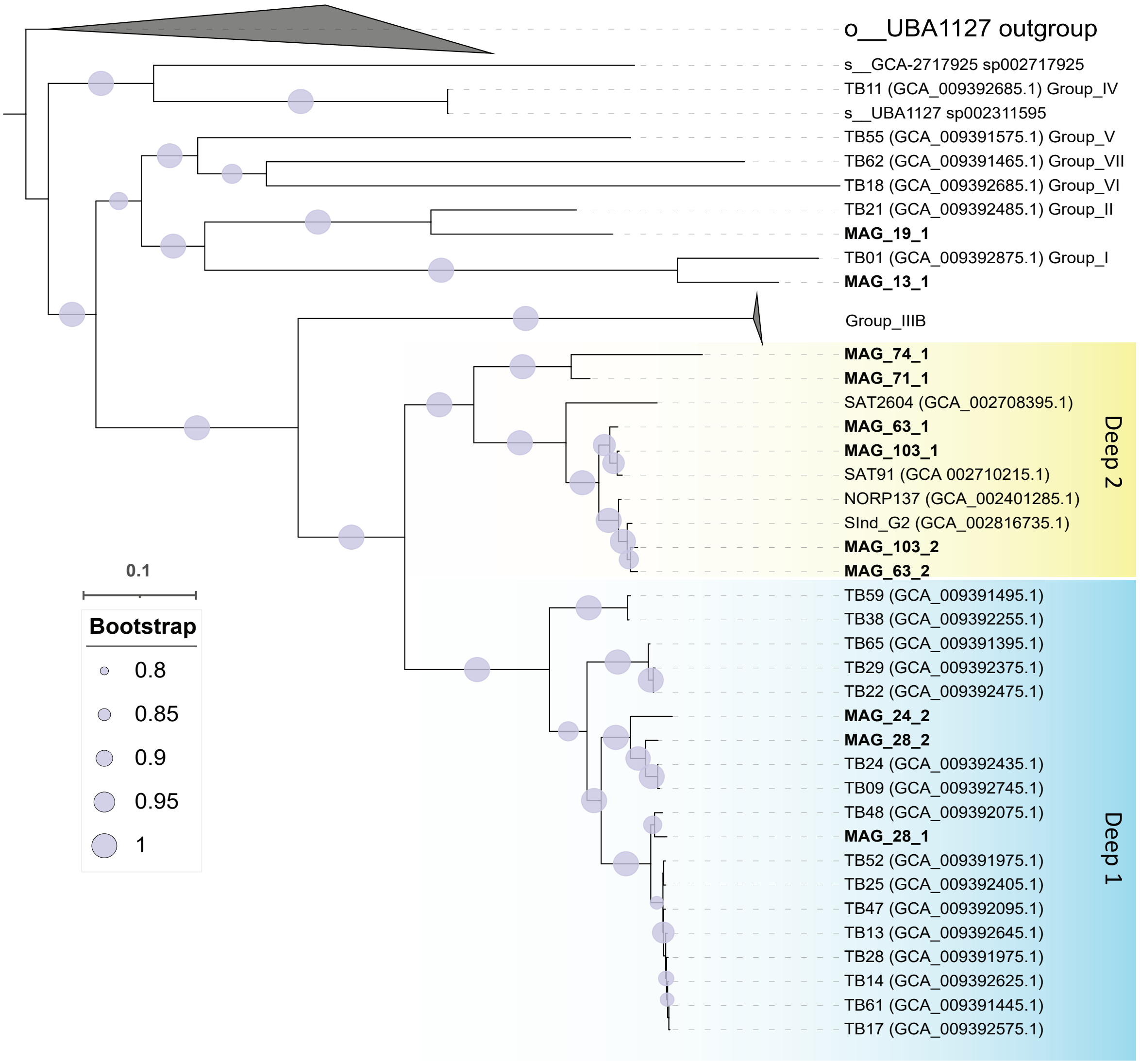

B)

	MAG_28_1	MAG_28_2	TB65_GroupIII	MAG_103_1	MAG_103_2
MAG_28_1	*	79.26	80.15	69.36	69.68
MAG_28_2	79.55	*	80.32	69.61	69.88
TB65_GroupIII	79.2	79.4	*	69.38	69.36
MAG_103_1	69.17	69.71	69.85	*	87.04
MAG_103_2	69.56	69.79	69.76	86.33	*

Figure S5: Phylogenetic analysis of SAR202 genomes, focusing on Group_IIIA (Deep 1 and Deep 2). A) The phylogenetic tree is based on an alignment of 120 bacterial marker genes from published SAR202 MAGs of different groups and MAGs retrieved in this study. MAGs retrieved in this study are given in bold. The tree was calculated using GTDB-Tk (https://github.com/Ecogenomics/GtdbTk). B) Average nucleotide identity between SAR202 MAGs recovered in this study and the representative of Group IIIA (Deep 1).

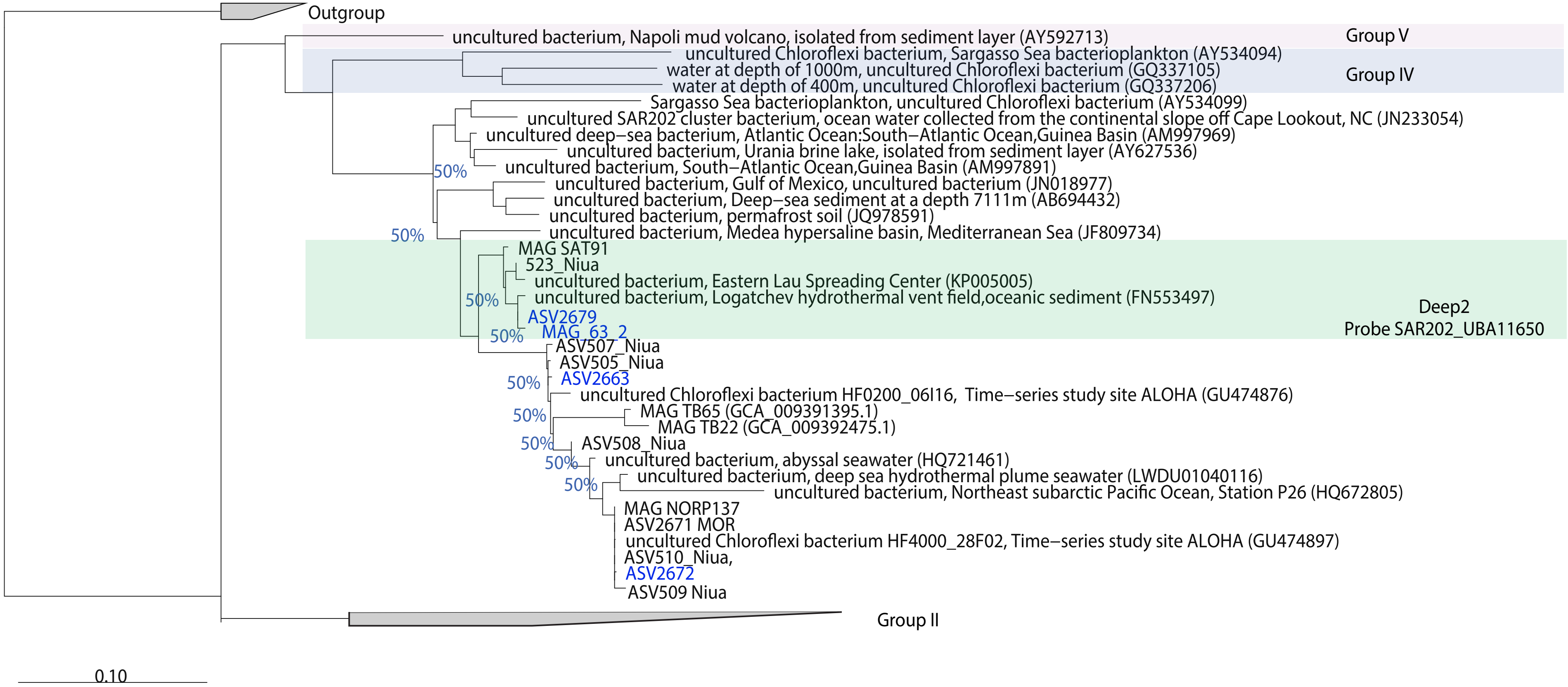

Figure S6: Phylogenetic tree of the SAR202 clade based on 16S rRNA gene sequences. This consensus tree was calculated based on 92 long sequences using PhyML and RaxML trees, with a 30% sequence conservation filter, and 100 bootstraps. 16S rRNA gene sequences of published MAGs (e.g. TB65) are added to the tree. ASVs retrieved in this study are named ASV[number], while the ASV retrieved from Niua South are named ASV[number]_Niua. The green bar represents the sequences targeted by probe SAR202_UBA11650. The bar indicates 10% estimated sequence changes.

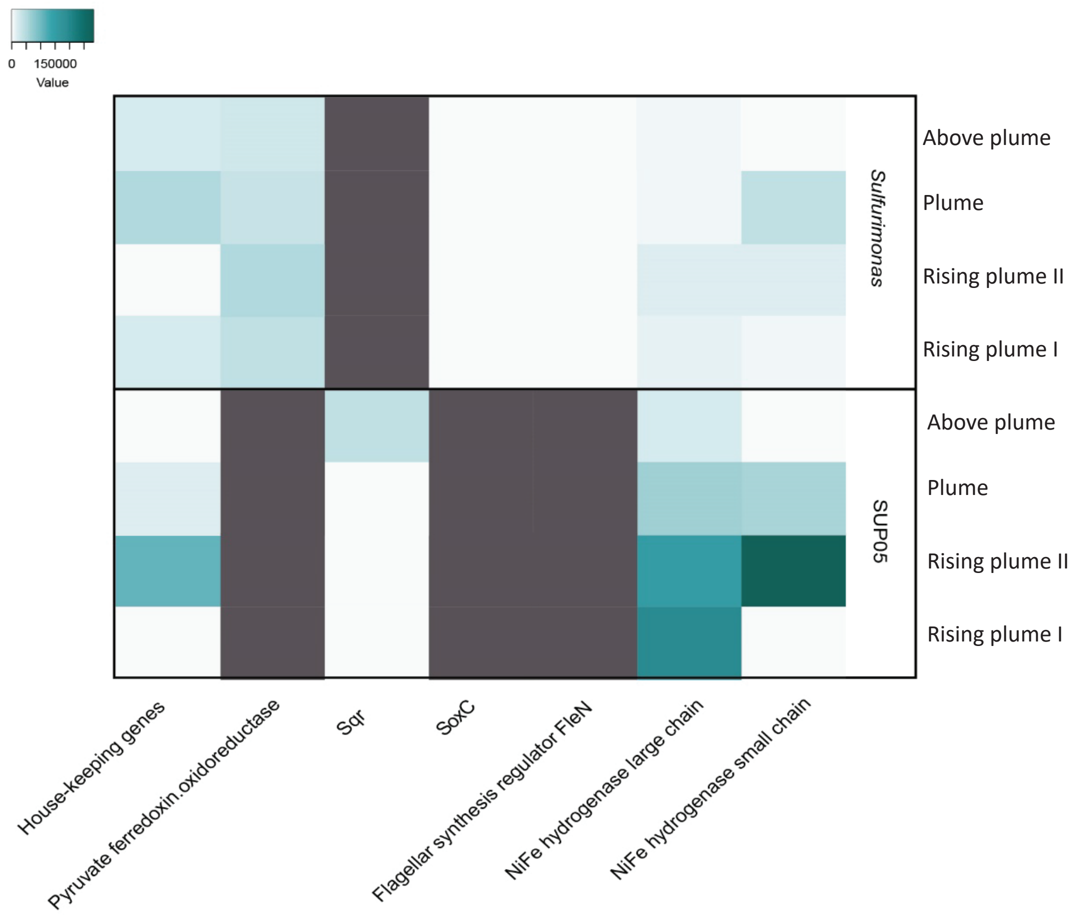

Figure S7: Expression of pyruvate:ferredoxin oxidoreductase (rTCA), hydrogenase genes, Sulfide:quinone oxidoreductase, *soxC* and flagella in Sulfurimonas and SUP05. Metatranscriptomes of Von Damm from 2 rising plume (rising plume I - SRR2044842; rising plume II - SRR2044843) and 2 plume samples (plume - SRR2044878; Above plume - SRR2044888) were mapped to MAG_107 (*Sulfurimonas*) and MAG_65_1 (SUP05) with 97% identity. Normalized abundance of transcripts is given in transcripts per million reads (TPM).

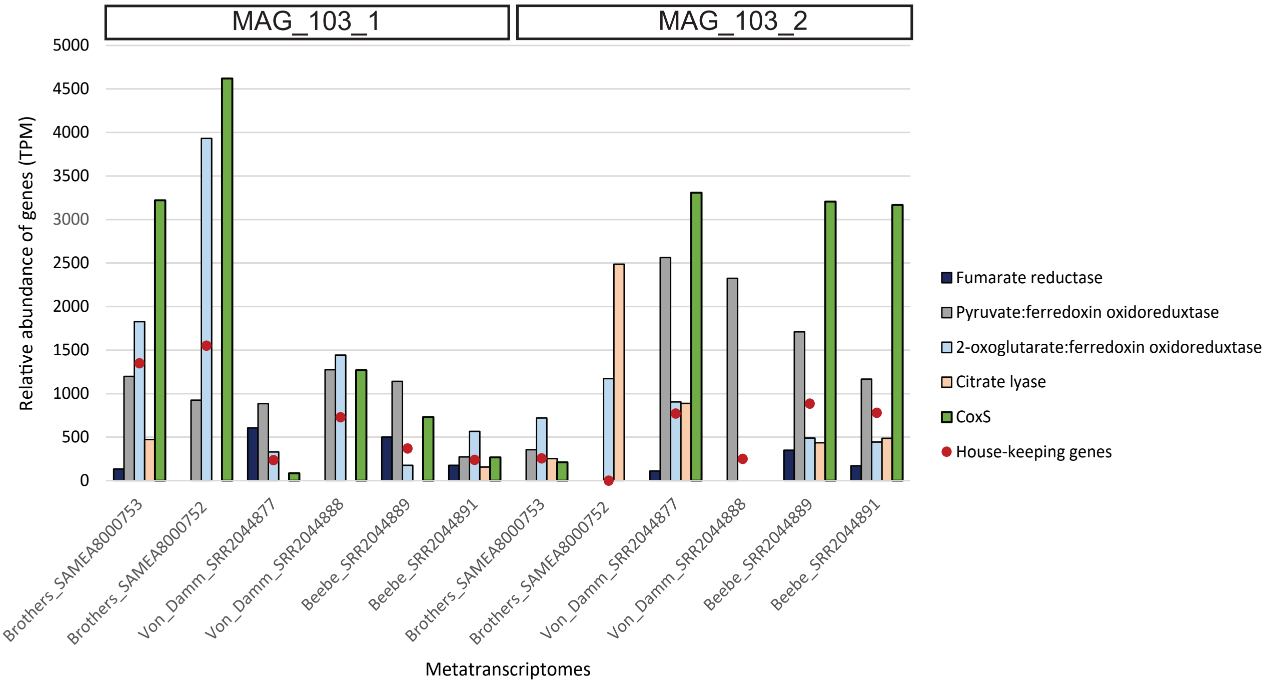

Figure S8: Expression of rTCA key gene, CO dehydrogenase and house-keeping genes in SAR202 MAGs: MAG_103_1 and MAG_103_2. Metatranscriptomes of Brothers, Von Damm and Beebe were mapped to MAGs with 97% identity. Expression was given in TPM.

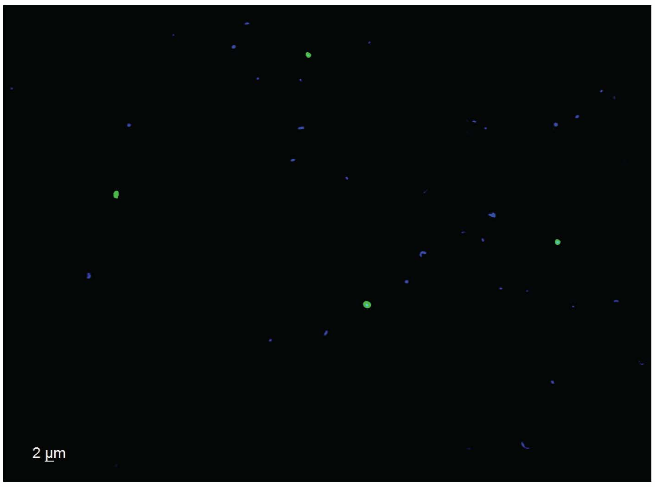

Figure S9: Photo of *Carboxydicoccus* cells in Iri_Site2_rp. CARD-FISH was conducted using SAR202_UBA11650 probe and DAPI for DNA staining. Blue: DAPI staining. Green: CARD-FISH staining.

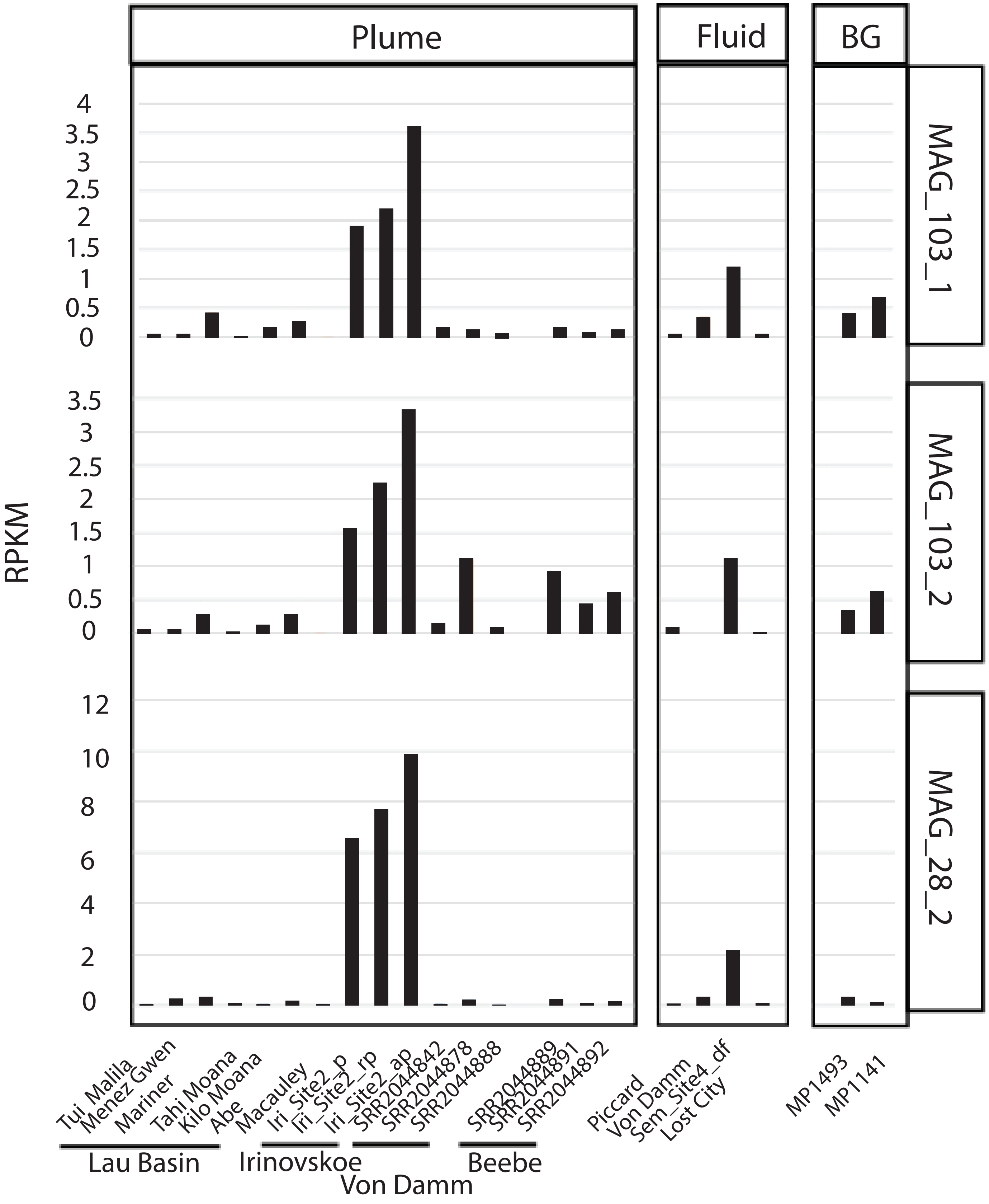

Figure S10: Abundance of MAG_103_1, MAG_103_2 and MAG_28_2 in plumes (Lau Basin, Irinovskoe, Von Damm and Beebe), fluid and diffusive fluids (Piccard, Von Damm, Semenov II and Lost City) and background samples at 3000-4000 m depth. Abundance is given in RPKM.

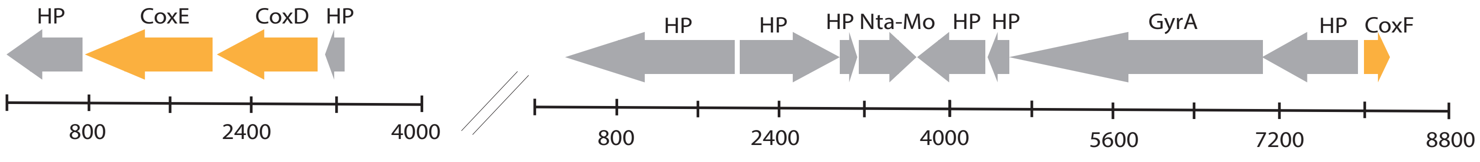

Figure S11: Arrangement of Cox accessory genes in MAG_103_1 across two contigs. Accessory genes are given in orange, whereas other genes are in gray.
