# Supplementary material for: Bacterial chemolithoautotrophy in ultramafic plumes along the Mid-Atlantic Ridge
Source: ISME J. 2024 Aug 20;18(1):wrae165. doi: 10.1093/ismejo/wrae165 (PMC11411561; doi:10.1093/ismejo/wrae165)
Supplement: Supplementary_Information_BD_wrae165 [file supplementary_information_bd_wrae165.pdf]

# Bacterial chemolithoautotrophy in ultramafic plumes along the Mid-Atlantic Ridge

Bledina Dede[1‡*], Eoghan P. Reeves[2], Maren Walter[3,4], Wolfgang Bach[4,5], Rudolf Amann[1], Anke Meyerdierks[1*]

[1] Max Planck Institute for Marine Microbiology, Bremen, Germany

[2] Department of Earth Science and Centre for Deep Sea Research, University of Bergen, Bergen, Norway

[3] Institute of Environmental Physics, University of Bremen, Bremen, Germany

[4] MARUM, Center for Marine Environmental Sciences, University of Bremen, Bremen, Germany

[5] Geoscience Department, University of Bremen, Bremen, Germany

‡ present address: Ecologie Systématique Evolution, CNRS, Université Paris-Saclay, AgroParisTech, Gif-sur-Yvette, France

# Supplementary Information

## Suppplementary methods

### Helium isotope analysis

Noble gas samples, for subsequent analysis in the home laboratory, were taken with the CTD water sampler to determine the concentrations of primordial helium (He) in the plume. Individual samples were drawn directly after recovery into gas-tight copper tubes, avoiding air

contamination by gas bubbles. Samples were analyzed for content of $^3He$ and $^4He$ isotopes at the mass spectrometric facility of the University of Bremen (Germany) with a combination of a high-resolution sector field mass spectrometer and a quadrupole mass spectrometer (1). Total errors based on the instrument performance and standard reference samples for $^3He$, $^4He$, and Ne concentrations were below 2%, 1%, and 1%, respectively. Hydrothermally sourced primordial He is characterized by an excess of the isotope 3He. Thus, values are reported as δ3 He (in %), defined as (3He/4He)/Ra – 1, the fractional deviation of the 3He/4He ratio in samples from that of air (Ra = 1.384 × 10−6).

## Thermodynamic calculations

Standard Gibb's energy (ΔrGo) of aerobic $H_2$ and $H_2S$ oxidation was calculated using SUPCRT92 for a reference state of 2.5°C and 30 MPa. The calculations of ΔrG were done as a function of plume dilutions (up to $1{:}10^6$ fluid:seawater) (Table 3). Constant activities of $O_2$(aq): 250µM, sulfate: 3 mM, and $H^+$: pH of 8.0 were assumed.

## Catalyzed reporter deposition-FISH (CARD-FISH)

Probes used were EUBI-III mix (2,3), NON338 (4), ARCH915 (5), SAR202_312R (6), a mixture of EPS549 and EPS682 (7,8), and SUP05_1241 (9). Samples targeted with ARCH915 for the visualization of Archaea were additionally permeabilized with 15 µg/mL proteinase K for 3 minutes.

A new probe was designed in order to visualize and enumerate the SAR202 genus UBA11650. The probe SAR202_UBA11650 (5'-CTGCCCTCAAGCCTGGTAGTAT-3') had 0 mismatches to the target sequences available in the SILVA database and only two outgroup hits in *Halobacterales* and *Bathyarchaeota*. Two helper oligonucleotide probes were designed (Helper1: 5'-

AATTCCGCCTTCCTCTA-3’; Helper2: 5’-TCCCTTGACCTCTCCCAG-3’) (10). The probes were hybridized using a 25% formamide concentration at 46 $^{o}$C. A Nikon Eclipse 50i microscope was used for taking photos and quantification.

## MAG analysis

The estimation of MAGs’ contamination and completeness was done by CheckM v2 (11). Additionally, the abundance of MAGs was assessed for Irinovskoe and Semenov-2 metagenomes through read recruitment using BBMap (99% identity) (12), and subsequent conversion of values to reads per kilobase per million (RPKM).

Average nucleotide identity (ANI) was calculated using JSpeciesWS v3.9.0 (13). A genome-based phylogenetic tree was calculated using 120 bacterial marker genes in GTDB-Tk v1.3.0 using GTDB r214 database (14) and other published MAGs (15). PacBio 16S rRNA gene amplicon sequences, 16S rRNA gene sequences retrieved from MAGs and a selection of previously published sequences were added to the reference phylogenetic tree of the SILVA SSU Ref138 NR99 database in ARB (16,17). De novo phylogenetic trees were calculated for clades of interest using PhyML (18) and RaxML (19) algorithms with different bacterial position variability filters (20, 25, 30 and 50%). A consensus tree was finally calculated based on PhyML and RaxML trees in ARB. MAGs were annotated as previously described (20). Iron related genes were checked using FeGenie v1.2 (21).

A phylogenetic tree was also constructed using translated genes encoding the CODH large subunit gene (*CoxL*) retrieved from SAR202 MAG_103_1 and MAG_103_2 and 63 sequences of the UniProtKB database (22). Sequences were aligned using MUSCLE (23) and the tree was calculated with FastTree (24).

# Supplementary results

## Microbial community composition

Based on 16S rRNA gene amplicons, the plume originating from Irinovskoe (Fig. 1) was dominated by chemolithoautotrophic *Sulfurimonas* (*Campylobacterota*; up to 23%) and SUP05 bacteria (*Thioglobaceae*; up to 40%). In Irinovskoe, SUP05 was higher in abundance in the rising plume sample, whereas *Sulfurimonas* dominated the plume samples. Additionally, among the highly abundant bacteria were deep-sea typical clades, including SAR202 (*Chloroflexaeota*; up to 10%), SAR406 (*Marinimicrobia*; up to 11%), as well as *Planctomycetota* (up to 7%).

Samples of Semenov-2 were retrieved from the fluid and diffuse fluid above mussel field. The fluid sample was dominated by chemolithoautotrophic *Sulfurimonas* (36%) and SUP05 (28%) clades. The deep-sea typical clades were either not detected (SAR202), or low in abundance (4% - SAR406). In the diffuse fluid above the mussel field, *Sulfurovum* (11%), SAR202 (10%), SAR406 (14%), SAR324 (7%), and SUP05 (3%) were the most prominent taxa within the community.

The fluid of Ashadze-2 was rich in *Sulfurimonas* (17%) and SUP05 (17%). Similarly to Irinovskoe, *Sulfurimonas* (up to 66%) and SUP05 (up to 20%) dominated the plume community. Other abundant bacteria affiliated with SAR406 (11%), SAR202 (5%), and *Alteromonas* (7%).

*Sulfurimonas* also dominated the Logatchev-1 plume community, reaching up to 51% relative abundance. As in other analyzed plumes, SUP05 was the second most abundant group of chemolithoautrophic bacteria (up to 14% - LHF_Site1_p). Other abundant bacteria were SAR406 (8% - LHF_Site1_bp), SAR202 (7% - LHF_Site1_bp), and SAR324 (8% - LHF_Site1_bp). The community of the diffuse fluid above the mussel field was rich in *Sulfurovum* (20%), similarly to Semenov-2's community.

### Single-nucleotide variation calculations

Synonymous (S) and nonsynonymous (N) mutations in MAG_103_1 and MAG_103_2 were determined through SNV patterns in plume and background metagenomes (2000-3000 m depth). SNV counts and N/S SNV of MAG_103_1 and MAG_103_2 were higher in plumes than in the deep-sea metagenomes. N/S SNVs were <1, indicating that the genes were under stabilizing conditions and mutations were biased toward the synonymous types (Table S7).

## Taxonomic descriptions

New taxon has been described under the SeqCode (25):

***Carboxydicoccus* gen. nov. Etymology.** *Carboxydum* (N.L. neut. adj.): carbon monoxide*;* *kokkos* (Gr. masc. n.): grain; *Carboxydicoccus* (N.L. masc. n.): utilizing carbon monoxide and grain looking like. Type species***: Carboxydicoccus profundi***.

***Carboxydicoccus profundi* sp. nov. Etymology**. *Profundi* (L. gen. n.): of the depths, living within the depths of the oceans.

**Diagnosis**. This bacterium lives in hydrothermal plumes of the deep-sea. It has a genome of around 3.1 Mbp (GC content: 57.5%). It is known from environmental sequencing. Using a specific probe - SAR202_UBA11650 (5'-CTGCCCTCAAGCCTGGTAGTAT-3') with helpers (Helper1: 5'-AATTCCGCCTTCCTCTA-3'; Helper2: 5'-TCCCTTGACCTCTCCCAG-3'), species of ***Carboxydicoccus* gen. nov.** were targeted and visualized. These cells have an average diameter of 1-1.5µm. The designated type MAG is SAT91 (GCA_002710215), which was obtained from a co-assembly of Tara metagenome South Atlantic. It has a higher abundance in the mesopelagic TARA samples. It

harbors genes for carbon fixation via rTCA cycle and chemolithotrophy fueled by aerobic oxidation of carbon monoxide.

***Thioglobus ultramaficus* sp. nov. Etymology**. *ultramaficus* (N.L. masc. adj.): living in the plumes released in ultramafic-hosted systems.

**Diagnosis**. This bacterium lives in hydrothermal plumes of the deep-sea. It has a genome of around 1.2 Mbp (GC content: 38.1%). It is known from environmental sequencing. It harbors genes for carbon fixation via CBB cycle and genes for sulfur and hydrogen oxidation.

## Supplementary tables

**Table S1: Metagenomic information on three Irinovskoe samples and one Semenov-2 sample.** Genomes estimation (GE) was determined by calculating the average sequencing depth of 16 universal, single-copy ribosomal protein genes per sample, yielding counts on a "per genome" basis following Microbe Census *(26)*. Samples were sequenced using the Illumina HiSeq2500 technology.

| | Raw reads | Nonpareil (%) | Assembly | | |
|---|---|---|---|---|---|
| | | | contigs (>5000 kb) | GC (%) | Genome estimation |
| Iri_Site2_p | 3.14E+07 | 54 | 3963 | 50.9 | 4921.88 |
| Iri_Site2_rp | 2.62E+07 | 58 | 1522 | 50.39 | 3596.27 |
| Iri_Site2_ap | 1.09E+07 | 54 | 1283 | 50.67 | 5118.32 |
| Sem_Site4_df | 2.62E+07 | 55 | 8867 | 50.45 | 3208.21 |

**Table S2: List of metagenomes and metatranscriptomes recruited onto MAGs retrieved in this study.** BBMap *(12)* was used with 99% identity filter for metagenomes and 97% identity filter for metatranscriptomes.

| Location | Dataset | Accession | Characteristic | Reference |
|---|---|---|---|---|
| TARA Oceans | | PRJEB1787 | Surface, DCM, mesopelagic | (27) |
| Malaspina | Metagenome | -- | Deep-sea | (28) |
| Macauley | Metagenome | PRJEB42974 | Sulfur-rich | (20) |
| Brothers | Metagenome | PRJEB42974 | Sulfur-rich | (20) |
| Mariner | Metagenome | PRJNA234377 | Sulfur-rich | (29) |
| Tui Malilla | Metagenome | PRJNA234377 | Sulfur-rich | (29) |
| Woody Crack | Metagenome | PRJEB11362 | Methane-rich | (9) |
| Brothers | Metatranscriptome | PRJEB42974 | Sulfur-rich | (20) |
| Von Damm | Metatranscriptome | PRJNA283173 | Hydrogen-rich | (30) |
| Beebe (Piccard) | Metatranscriptome | PRJNA283173 | Hydrogen-rich | (30) |
| Lost City fluid | Metagenome | PRJNA779602 | Hydrogen-rich | (31) |
| Von Damm | Metagenome | PRJEB9204 | Hydrogen-rich | (32) |

| | | | | |
|---|---|---|---|---|
| Piccard | Metagenome | PRJEB9204 | Hydrogen-rich | (32) |

**Table S3: MAGs retrieved in this study.** Quality of the MAGs was assessed based on completeness and contamination. MAGs were dereplicated with >95% average nucleotide identity (ANI), with the exception of *Sulfurimonas*, *Sulfurovum* and SAR202 MAGs. Abundance of MAGs which share >95% ANI has given only for the most complete MAG. Relative abundance of MAGs in each metagenome was evaluated using a 99% identity threshold with BBMap. MAGs were taxonomically classified using GTDB-tk.

| | | Quality | | | Abundance RPKM | | | |
|---|---|---|---|---|---|---|---|---|
| MAG | Taxonomy | Complet. | Contam. | Length | Iri_Site2_p | Iri_Site2_rp | Iri_Site2_ap | Sem_Site4_df |
| MAG_65_1 | SUP05 | 97.02 | 0.66 | 1190886 | 5.06 | 3.24 | 0.81 | 0.36 |
| MAG_2_2 | *Sulfurimonas* | 72.63 | 2.87 | 1095587 | 5.72 | 2.00 | 0.47 | 0.52 |
| MAG_107 | *Sulfurimonas* | 82.34 | 3.5 | 1241242 | | | | |
| MAG_17_2 | *Sulfurimonas* | 89.71 | 4.02 | 1419594 | 7.57 | 2.49 | 0.57 | 0.64 |
| MAG_104 | *Sulfurovum* | 71.98 | 5.22 | 1603663 | | | | |
| MAG_31 | *Sulfurovum* | 60.02 | 0.9 | 1373210 | 0.31 | 0.01 | 0.00 | 1.1 |
| MAG_71_1 | SAR202 | 43.75 | 7.62 | 1996286 | 1.36 | 1.66 | 2.18 | 0.58 |
| MAG_24_2 | SAR202 | 84.23 | 0.50 | 2817100 | 6.72 | 7.84 | 8.99 | 1.97 |
| MAG_13_1 | SAR202 | 73.05 | 3.26 | 1773562 | 0.95 | 1.14 | 0.95 | 0.38 |
| MAG_74_1 | SAR202 | 53.96 | 7.51 | 3883610 | 1.10 | 1.16 | 1.50 | 0.38 |
| MAG_19_1 | SAR202 | 73.83 | 1.98 | 2661864 | 0.96 | 1.32 | 0.84 | 0.54 |
| MAG_63_1 | SAR202 | 40.99 | 8.17 | 1689757 | | | | |
| MAG_103_1 | SAR202 | 67.89 | 5.74 | 2268886 | 1.88 | 2.18 | 3.6 | 1.19 |
| MAG_28_1 | SAR202 | 66.12 | 1.19 | 1892302 | 6.53 | 7.44 | 10.5 | 1.89 |
| MAG_63_2 | SAR202 | 66.81 | 10.65 | 3277150 | | | | |
| MAG_103_2 | SAR202 | 63.14 | 7.48 | 2762128 | 1.55 | 2.22 | 3.33 | 1.11 |
| MAG_28_2 | SAR202 | 60.82 | 4.95 | 1991698 | 6.47 | 7.65 | 9.82 | 2.1 |
| MAG_49_1 | *Poseidoniia* | 63.35 | 4.13 | 1504548 | 0.88 | 0.35 | 1.09 | 0.18 |
| MAG_32_2 | *Poseidoniia* | 62.25 | 6.40 | 1069430 | 2.17 | 1.21 | 4.71 | 0.77 |
| MAG_138 | *Poseidoniia* | 70.17 | 5.07 | 1295131 | 2.73 | 1.43 | 6.56 | 0.94 |
| MAG_70_1 | *Poseidoniia* | 59.56 | 2.40 | 1206495 | 2.45 | 1.28 | 3.47 | 0.68 |
| MAG_158_1 | *Poseidoniia* | 52.06 | 6.49 | 663347 | 1.73 | 1.46 | 2.95 | 0.75 |
| MAG_81_7 | Archaea | 78.01 | 9.30 | 795945 | 5.75 | 5.30 | 3.75 | 1.56 |
| MAG_125_1 | Planctomyteota | 92.28 | 1.61 | 2780124 | 2.35 | 2.16 | 1.24 | 0.70 |
| MAG_32_1 | Planctomyteota | 58.14 | 1.85 | 992651 | 0.66 | 0.39 | 0.77 | 0.22 |
| MAG_88_1 | Planctomyteota | 53.86 | 1.26 | 868614 | 2.80 | 1.48 | 6.93 | 1.00 |

| | | | | | | | | |
|---|---|---|---|---|---|---|---|---|
| MAG_136_1 | *Acidimicrobia* | 57.33 | 2.14 | 1305163 | 0.53 | 0.48 | 0.43 | 0.87 |
| MAG_63_1 | *Acidimicrobia* | 81.89 | 5.12 | 2209050 | 0.74 | 0.72 | 0.75 | 1.84 |
| MAG_158 | Myxococcota | 51.96 | 4.09 | 2293569 | 0.52 | 0.37 | 0.29 | 0.10 |
| MAG_89 | Myxococcota | 63.05 | 3.71 | 2861545 | 0.51 | 0.41 | 0.26 | 0.09 |
| MAG_150_1 | SAR324 | 50.3 | 5.61 | 1435292 | 3.47 | 2.50 | 4.35 | 2.02 |
| MAG_7_1 | Verrucomicrobiota | 69.66 | 8 | 2080070 | 4.23 | 4.52 | 5.80 | 1.06 |
| MAG_19_1 | *Alteromonas* | 52.12 | 4.1 | 1939763 | 0.13 | 0.09 | 1.61 | 12.30 |
| MAG_78_1 | *Alteromonas* | 97.87 | 4.08 | 4458181 | 0.01 | 0.01 | 0.09 | 1.76 |
| MAG_78_3 | *Alteromonas* | 80.66 | 10.74 | 3797918 | 0.01 | 0.01 | 0.27 | 2.11 |
| MAG_78_2 | *Alteromonas* | 63.24 | 7.76 | 3289841 | 0.01 | 0.01 | 0.15 | 1.83 |
| MAG_56 | *Marinobacter* | 84.92 | 2.5 | 3274144 | 0.58 | 0.12 | 0.11 | 0.44 |
| MAG_10_2 | *Idiomarina* | 95.83 | 2.47 | 2548562 | 0.02 | 0.29 | 0.03 | 1.23 |
| MAG_51 | *Ketobacter* | 88.89 | 5.79 | 4419946 | 0.00 | 0.00 | 0.01 | 0.57 |
| MAG_83_1 | *Alcanivorax* | 75.46 | 1.71 | 2464085 | 0.10 | 0.08 | 1.24 | 3.80 |
| MAG_45 | *Alcanivorax* | 69.49 | 4.12 | 1993666 | 0.19 | 0.20 | 0.28 | 0.97 |
| MAG_29_1 | *Halomonas* | 72.54 | 2.83 | 1980216 | 0.38 | 0.05 | 0.62 | 0.72 |
| MAG_165_1 | *Pseudomonales* | 57.74 | 2.16 | 1649649 | 0.54 | 0.28 | 1.05 | 0.44 |
| MAG_123_1 | *Acinetobacter* | 67.47 | 2.51 | 1896727 | 0.02 | 0.01 | 0.04 | 1.39 |
| MAG_96 | *Erythrobacter* | 70.58 | 2.53 | 2247043 | 0.29 | 0.50 | 0.59 | 0.66 |
| MAG_162 | *Methylococcales* | 69.79 | 2.53 | 1361923 | 0.40 | 0.34 | 0.30 | 0.74 |
| MAG_115_1 | *Marinisomatales* | 52.56 | 1.65 | 1072186 | 3.41 | 2.11 | 2.66 | 1.58 |

**Table S4: Comparison of the abundance of MAG 65_1 and *Thioglobus vulcanius* (20) in plumes of Brothers and Macauley vents.** Abundance is given in RPKM.

| Kermadec Arc | Sample | MAG_65_1 | *Thioglobus vulcanius* |
|---|---|---|---|
| Macauley | 04CTD_b6 | 0.0 | 0 |
| Macauley | 10CTD_b2 | 0.0 | 0.1 |
| Macauley | 10CTD_b4 | 0.0 | 0.1 |
| Macauley | 10CTD_b10 | 0.0 | 0 |
| Macauley | 10CTD_b12 | 0.0 | 0 |
| Brothers | 49CTD_b10 | 1.0 | 8.3 |

| | | | |
|---|---|---|---|
| Brothers | 54CTD_b8 | 0.7 | 49.9 |
| Brothers | 54CTD_b12 | 0.3 | 25 |
| Brothers | 49CTD_b16 | 0.1 | 7.2 |

**Table S5: RPKM abundance of MAG_24_2, MAG_103_1 and MAG_103_2 (SAR202), MAG_17_2 (*Sulfurimonas*) and MAG_65_1 (SUP05) in bathypelagic metagenomes of Malaspina datasets.** Reads were mapped to the MAGs using BBMap with 99% minimum identity. Abundance was calculated as RPKM.

| | | | | SAR202 | | *Sulfurimonas* | SUP05 |
|---|---|---|---|---|---|---|---|
| | Longitude | Latitude | Depth | MAG_103_1 | MAG_103_2 | MAG_17_2 | MAG_65_1 |
| MP0145 | -26 | 14.52 | 4005.01 | 2.354 | 2.486 | 0.004 | 0.078 |
| MP0371 | -33.41 | -15.83 | 4003.17 | 1.337 | 1.401 | 0.002 | 0.013 |
| MP0372 | -33.41 | -15.83 | 4003.17 | 4.139 | 4.359 | 0.003 | 0.002 |
| MP0103 | -23.45 | 21.51 | 4005.12 | 3.741 | 3.940 | 0.003 | 0.022 |
| MP0203 | -26 | 7.33 | 4002.51 | 1.809 | 1.941 | 0.005 | 0.047 |
| MP0326 | -30.19 | -9.12 | 4001.48 | 0.977 | 1.043 | 0.002 | 0.017 |
| MP0262 | -27.33 | -3.03 | 4002.04 | 1.337 | 1.420 | 0.003 | 0.014 |
| MP0900 | 39.89 | -33.55 | 4002.12 | 0.335 | 0.376 | 0.003 | 0.037 |
| MP0901 | 39.89 | -33.55 | 4002.12 | 0.181 | 0.233 | 0.006 | 0.059 |
| MP0740 | 6.84 | -31.81 | 4001.3 | 2.482 | 2.614 | 0.003 | 0.069 |
| MP0758 | 12.77 | -32.81 | 3901.64 | 0.506 | 0.546 | 0.002 | 0.039 |
| MP0759 | 12.77 | -32.81 | 3901.64 | 1.533 | 1.627 | 0.005 | 0.233 |
| MP0555 | -21.43 | -26.91 | 3199.21 | 0.570 | 0.582 | 0.002 | 0.013 |
| MP0556 | -21.43 | -26.91 | 3199.21 | 3.803 | 3.913 | 0.004 | 0.019 |
| MP0626 | -11.8 | -28.62 | 3850.45 | 0.490 | 0.499 | 0.003 | 0.021 |
| MP0739 | 6.84 | -31.81 | 4001.3 | 0.406 | 0.422 | 0.001 | 0.010 |
| MP0959 | 63.25 | -27.98 | 3504.69 | 0.201 | 0.222 | 0.463 | 0.032 |
| MP1092 | 82.62 | -29.81 | 4000.29 | 2.740 | 2.850 | 0.006 | 0.025 |
| MP1140 | 92.99 | -29.65 | 2402.15 | 0.292 | 0.270 | 0.006 | 0.006 |
| MP1141 | 92.99 | -29.65 | 2402.15 | 0.673 | 0.625 | 0.013 | 0.030 |
| MP1202 | 103.31 | -30.33 | 4000.85 | 2.567 | 2.713 | 0.004 | 0.015 |
| MP1374 | 135.19 | -39.23 | 3995.55 | 1.355 | 1.449 | 0.006 | 0.022 |
| MP1896 | -150.35 | 21.06 | 4012.79 | 0.229 | 0.255 | 0.000 | 0.004 |
| MP1434 | 150.41 | -38.64 | 4001.15 | 0.405 | 0.429 | 0.001 | 0.026 |

| | | | | | | | |
|---|---|---|---|---|---|---|---|
| MP1482 | 179.14 | -28.41 | 3501.1 | 0.367 | 0.380 | 0.001 | 0.010 |
| MP1483 | 179.14 | -28.41 | 3501.1 | 1.926 | 1.946 | 0.002 | 0.065 |
| MP1493 | -179.52 | -25.49 | 2147.76 | 0.350 | 0.329 | 0.000 | 0.006 |
| MP1648 | -170.77 | -5.74 | 4017.73 | 0.267 | 0.296 | 0.001 | 0.009 |
| MP1649 | -170.77 | -5.74 | 4017.73 | 2.227 | 2.313 | 0.004 | 0.032 |
| MP2016 | -133.26 | 18.04 | 4004.03 | 3.513 | 3.520 | 0.001 | 0.009 |
| MP2052 | -124.49 | 15.91 | 4002.4 | 0.570 | 0.579 | 0.001 | 0.007 |
| MP2158 | -108.06 | 12 | 3103.11 | 0.516 | 0.528 | 0.001 | 0.027 |
| MP2159 | -108.06 | 12 | 3103.11 | 0.826 | 0.851 | 0.002 | 0.244 |
| MP2252 | -99.25 | 10.09 | 3007.91 | 0.119 | 0.156 | 0.002 | 0.012 |
| MP2253 | -99.25 | 10.09 | 3007.91 | 1.330 | 1.372 | 0.003 | 0.113 |
| MP2914 | -23.69 | 29.97 | 4003.49 | 0.139 | 0.170 | 0.004 | 0.025 |
| MP2633 | -52.63 | 20 | 4002.69 | 0.779 | 0.820 | 0.002 | 0.009 |
| MP2634 | -52.63 | 20 | 4002.69 | 4.102 | 4.299 | 0.002 | 0.009 |
| MP2968 | -17.26 | 32.08 | 4002.6 | 0.311 | 0.334 | 0.001 | 0.011 |

**Table S6: Digital Protologue for *Carboxydicoccus* gen.nov. and the new SUP05 species.**

| **Genus name** | *Carboxydicoccus* | *Thioglobus* |
|---|---|---|
| **Type species name** | *Carboxydicoccus profundi* sp. nov. | *Thioglobus ultramaficus* sp. nov. |
| **Specific epithet** | profundi | ultramaficus |
| **Genus status** | gen. nov. | sp. nov. |
| **Status** | uncultivated | uncultivated |
| **Etymology** | *Carboxydicoccus - N.L. neut. adj. carboxydum, carbon monoxide; Gr. masc. n. kokkos, grain; N.L. masc. n. Carboxydicoccus, utilizing carbon monoxide and grain looking like*<br><br>*Carboxydicoccus profundi* - L. gen. n. profundi, of the depths, living within the depths of the oceans | *Thioglobus ultramaficus*: L. adv. ultra, beyond; N.L. fem. adj. mafic, the term mafic which combines the first letters of 'magnesium' and 'ferrum' (iron); N.L. masc. adj. ultramaficus, referring to the fact that this bacterium was found in ultramafic hydrothermal plumes |
| **Description of the new taxon and diagnostic traits** | *Carboxydicoccus profundi* is abundant in above plume of Irinovskoe hydrothermal vent field in the Mid-Atlantic Ridge and the deep sea. The genome of the type strain was recovered from a co-assembly of South Atlantic Tara metagenomes.<br>Genome annotation predicts chemolithoautotrophy fueled by aerobic oxidation of carbon monoxide. Dark carbon fixation is conducted via rTCA cycle. | This bacterium lives in hydrothermal plumes of the deep-sea. It has a genome of around 1.2 Mbp (GC content: 38.1%). It is known from environmental sequencing. It harbors genes for carbon fixation via CBB cycle and genes for sulfur and hydrogen oxidation. |

| | | |
|---|---|---|
| **Country of origin** | Not applicable | Not applicable |
| **Region of origin** | South Atlantic | Mid-Atlantic Ridge |
| **Source of isolation** | South Atlantic – Tara Ocean metagenomes | Irinovskoe hydrothermal plume |
| **Sampling date (dd/mm/yyyy)** | 27/12/2016 | 29/04/2016 |
| **Latitude (xx°xx′xx″N/S)** | - | 13.33 N |
| **Longitude** | - | 44.91 W |
| **Depth (m)** | Mesopelagic and bathypelagic layer | 2653 |
| **Genome accession number** | GCA_002710215.1 | GCA_964016515.1 |
| **Genome status** | incomplete | incomplete |
| **Genome size** | 3074704 | 1190886 |
| **GC mol%** | 57.75 | 38.1 |
| **SeqCode registry URL (sp. nov.)** | seqco.de/r:7tg4nwu9 | |

**Table S7: Synonymous and Nonsynonymous Single nucleotide variation (SNV_S, SNV_N) in the plume and the deep-sea metagenomes of Malaspina of MAG_103_1 and MAG_103_2.** SNA were calculated using InStrain (33).

| | MAG_103_1 | | MAG_103_2 | |
|---|---|---|---|---|
| | Plume | Malaspina | Plume | Malaspina |
| SNV count / breadth minCov * length | 1.12E-02 | 2.66E-06 | 1.10E-02 | 1.49E-06 |
| SNV_S | 7.0 | 0.1 | 6.8 | 0.2 |
| SNV_N | 3.8 | 0.0 | 3.8 | 0.1 |
| N/S SNV | 0.6 | 0.3 | 0.5 | 0.4 |

# Reference


1. Sültenfuss J, Roether W, Rhein M. The Bremen mass spectrometric facility for the measurement of helium isotopes, neon, and tritium in water. Isotopes Environ Health Stud. 2009;45(2):83–95.

2. Amann RI, Binder BJ, Olson RJ, Chisholm SW, Devereux R, Stahl DA. Combination of 16S rRNA-targeted oligonucleotide probes with flow cytometry for analyzing mixed microbial populations. Appl Environ Microbiol. 1990;56(6):1919–25.

3. Daims H, Brühl A, Amann R, Schleifer KH, Wagner M. The domain-specific probe EUB338 is insufficient for the detection of all bacteria: Development and evaluation of a more comprehensive probe set. Syst Appl Microbiol. 1999;22(3):434–44.

4. Wallner G, Amann R, Beisker W. Optimizing fluorescent in situ hybridization with rRNA-targeted oligonucleotide probes for flow cytometric identification of microorganisms. Cytometry. 1993; 14(2):136-43.

5. Stahl D. A., Amann R. Development and application of nucleic acid probes in bacterial systematics. In: Stackebrandt E, Goodfellow M (eds). Nucleic Acid Techniques in Bacterial Systematics. 1991. John Wiley & Sons Ltd., Chichester, pp 205–248.

6. Morris RM, Rappé MS, Urbach E, Connon SA, Giovannoni SJ. Prevalence of the Chloroflexi-related SAR202 bacterioplankton cluster throughout the mesopelagic zone and deep ocean. Appl Environ Microbiol. 2004;70(5):2836–42.

7. Lin X, Wakeham SG, Putnam IF, Astor YM, Scranton MI, Chistoserdov AY, et al. Comparison of vertical distributions of prokaryotic assemblages in the anoxic cariaco basin and black sea by use of fluorescence in situ hybridization. Appl Environ Microbiol. 2006;72(4):2678-90.

8. Lin X, Scranton MI, Varela R, Chistoserdov A, Taylor GT. Compositional responses of bacterial communities to redox gradients and grazing in the anoxic Cariaco Basin. Aquat Microb Ecol. 2007;47(1).

9. Meier D V., Bach W, Girguis PR, Gruber-Vodicka HR, Reeves EP, Richter M, et al. Heterotrophic Proteobacteria in the vicinity of diffuse hydrothermal venting. Environ Microbiol. 2016;18(12):4348–68.

10. Fuchs BM, Glöckner FO, Wulf J, Amann R. Unlabeled helper oligonucleotides increase the in situ accessibility to 16S rRNA of fluorescently labeled oligonucleotide probes. Appl Environ Microbiol. 2000;66(8):3603–7.

11. Chklovski A, Parks DH, Woodcroft BJ, Tyson GW. CheckM2: a rapid, scalable and accurate tool for assessing microbial genome quality using machine learning. Nat Methods. 2023; 20(8):1203-1212.

12. Bushnell B. BBMap (version 35.14). 2015. Available at https://sourceforge.net/projects/bbmap/

13. Richter M, Rosselló-Móra R, Oliver Glöckner F, Peplies J. JSpeciesWS: A web server for prokaryotic species circumscription based on pairwise genome comparison. Bioinformatics. 2015;32(6):929–31.

14. Chaumeil PA, Mussig AJ, Hugenholtz P, Parks DH. GTDB-Tk: a toolkit to classify genomes with the Genome Taxonomy Database. Hancock J, editor. Bioinformatics. 2019;36(6):1925–7.

15. Saw JHW, Nunoura T, Hirai M, Takaki Y, Parsons R, Michelsen M, et al. Pangenomics analysis reveals diversification of enzyme families and niche specialization in globally abundant SAR202 bacteria. mBio. 2020;11(1):e02975-19.

16. Quast C, Pruesse E, Yilmaz P, Gerken J, Schweer T, Yarza P, et al. The SILVA ribosomal RNA gene database project: Improved data processing and web-based tools. Nucleic Acids Res. 2013;41(D1):D590-6.

17. Ludwig W, Strunk O, Westram R, Richter L, Meier H, Yadhukumar A, et al. ARB: A software environment for sequence data. Nucleic Acids Res. 2004;32(4):1363–71.

18. Guindon S, Dufayard JF, Lefort V, Anisimova M, Hordijk W, Gascuel O. New algorithms and methods to estimate maximum-likelihood phylogenies: Assessing the performance of PhyML 3.0. Syst Biol. 2010; 59(3):307–21.

19. Stamatakis A. RAxML version 8: A tool for phylogenetic analysis and post-analysis of large phylogenies. Bioinformatics. 2014;30(9):1312–3.

20. Dede B, Hansen CT, Neuholz R, Schnetger B, Kleint C, Walker S, et al. Niche differentiation of sulfur-oxidizing bacteria (SUP05) in submarine hydrothermal plumes. ISME J. 2022;16(6):1479-90.

21. Garber AI, Nealson KH, Okamoto A, McAllister SM, Chan CS, Barco RA, et al. FeGenie: A Comprehensive Tool for the Identification of Iron Genes and Iron Gene Neighborhoods in Genome and Metagenome Assemblies. Front Microbiol. 2020;11:499513

22. Magrane M, Consortium UP. UniProt Knowledgebase: A hub of integrated protein data. Database. 2011.

23. Edgar RC. MUSCLE: a multiple sequence alignment method with reduced time and space complexity. BMC Bioinform. 2004;5(1):113.

24. Price MN, Dehal PS, Arkin AP. FastTree 2 - Approximately maximum-likelihood trees for large alignments. PLoS one. 2010;5(3):e9490.

25. Hedlund, B.P., Chuvochina, M., Hugenholtz, P. *et al.* SeqCode: a nomenclatural code for prokaryotes described from sequence data. Nat Microbiol. 2022;7:1702–1708.

26. Nayfach S, Pollard KS. Average genome size estimation improves comparative metagenomics and sheds light on the functional ecology of the human microbiome. Genome Biol. 2015;16(1):1-18.

27. Sunagawa S, Coelho LP, Chaffron S, Kultima JR, Labadie K, Salazar G, et al. Structure and function of the global ocean microbiome. Science. 2015;348(6237):1261359.

28. Duarte CM. Seafaring in the 21st century: The Malaspina 2010 circumnavigation expedition. Limnol Oceanogr Bull. 2015;24:11–14.

29. Anantharaman K, Breier JA, Dick GJ. Metagenomic resolution of microbial functions in deep-sea hydrothermal plumes across the Eastern Lau Spreading Center. ISME J. 2016;10(1):225–39.

30. Li M, Baker BJ, Anantharaman K, Jain S, Breier JA, Dick GJ. Genomic and transcriptomic evidence for scavenging of diverse organic compounds by widespread deep-sea archaea. Nat Commun. 2015 61. 2015;6(1):8933.

31. Brazelton WJ, McGonigle JM, Motamedi S, Pendleton HL, Twing KI, Miller BC, et al. Metabolic Strategies Shared by Basement Residents of the Lost City Hydrothermal Field. Appl Environ Microbiol. 2022;88(17):e00929-22.

32. Reveillaud J, Reddington E, McDermott J, Algar C, Meyer JL, Sylva S, et al. Subseafloor microbial communities in hydrogen-rich vent fluids from hydrothermal systems along the Mid-Cayman Rise. Environ Microbiol. 2016;18(6):1970-87.

33. Olm MR, Crits-Christoph A, Bouma-Gregson K, Firek BA, Morowitz MJ, Banfield JF. inStrain profiles population microdiversity from metagenomic data and sensitively detects shared microbial strains. Nat Biotechnol. 2021;39(6):727–36.

34. Scilipoti S, Molari M. High-quality draft genome of Gammaproteobacterial SUP05 cluster from non-buoyant hydrothermal plumes of ultraslow spreading Gakkel Ridge (Central Arctic Ocean). Microbiol Resour Announc. 2024;13(4):e00121-24.
